# Global Distribution Patterns of Carbapenemase-Encoding Bacteria in a New Light: Clues on a Role for Ethnicity

**DOI:** 10.3389/fcimb.2021.659753

**Published:** 2021-06-29

**Authors:** Claudio Neidhöfer, Christian Buechler, Guido Neidhöfer, Gabriele Bierbaum, Irene Hannet, Achim Hoerauf, Marijo Parčina

**Affiliations:** ^1^ Institute of Medical Microbiology, Immunology and Parasitology, University Hospital Bonn, Bonn, Germany; ^2^ ZEW—Leibniz Centre for European Economic Research, Mannheim, Germany; ^3^ H.I.M.A. Consulting, Ninove, Belgium

**Keywords:** carbapenemases, carbapenem-resistant Enterobacterales, carbapenem-resistant *Acinetobacter baumannii*, carbapenem-resistant *Pseudomonas aeruginosa*, carbapenem-resistant Enterobacteriaceae, carbapenem-resistant Gram negative bacteria, antibiotic resistance screening

## Abstract

Antibiotic resistance represents a major global concern. The rapid spread of opportunistically pathogenic carbapenemase-encoding bacteria (CEB) requires clinicians, researchers, and policy-makers to swiftly find solutions to reduce transmission rates and the associated health burden. Epidemiological data is key to planning control measures. Our study aims to contribute by providing an analysis of 397 unique CEB isolates detected in a tertiary hospital in Germany. We propose new findings on demographic variables to support preventive sanitary precautions in routine clinical practice. Data on detected CEB was combined with patient’s demographic and clinical information for each isolate. Multiple regression techniques were applied to estimate the predictive quality of observed differences. Our findings confirm the role of age and gender in CEB colonization patterns and indicate a role for ethnicity and domicile. Also, carbapenemase-encoding *A. baumannii* was most frequently introduced to the hospital, while the risk of colonization with VIM-encoding *P. aeruginosa* rose with the length of hospital stay. *P. aeruginosa* remains an important complication of prolonged hospital stays. The strong link to hospital-wastewater may have implications for hospital-built environments. *A. baumannii* can be efficiently controlled from spreading at hospital admission. OXA-encoding CEB being harder to detect in routine screening, targeted preventive measures, such as culture media selective for carbapenem-resistant bacteria, would be opportune for patients from selected regions. The CEB differences linked to ethnicity found in our study may further be supporting the tailoring of diagnostic approaches, as well as health policies upon confirmation by other studies and a better understanding of their global distribution.

## Introduction

Bacteria excel at swiftly acquiring the genetic resources to thrive in environments that intend to inhibit their growth. Increasing anti-microbial resistance (AMR) continues to limit treatment options to where there may be no more cure. This growing threat to human health is a serious global concern with a significant health-economic burden. WHO considers the growing AMR issue one of the three major public health challenges of the 21st century, responsible for rising healthcare costs, extended hospital stays, treatment failures, and often death (Naylor et al., 2018; Dadgostar, 2019). The World Economic Forum, devoting a chapter to the growing public health challenge of AMR in its 2013 Global Risk Report, reported an intensified situation in 2018 and finally in 2020 predicted AMR to become the worldwide leading cause of death by 2050 if no action is taken.

Carbapenemase-encoding bacteria (CEB) have spread worldwide (Queenan and Bush, 2007; Lee et al., 2016; Logan and Weinstein, 2017). The most relevant carbapenemases include KPC, NDM, IMP, VIM, and OXA family enzymes, often carried on plasmids. Bacteria producing these enzymes are normally resistant not only to carbapenems but actually to nearly all antibiotics, often due to additional resistance genes carried on those plasmids (Johnning et al., 2018; Sawa et al., 2020). Previous studies have shown an overall rise of the prevalence of CEB in Europe and have highlighted the need for enhanced containment efforts at both country and European levels (Albiger et al., 2015; David et al., 2019).

Epidemiological data is of primary importance to understand the scope of the problem and to design effective control measures. We analyzed demographic and clinical data on 397 CEB detected at our University Hospital (Bonn, Germany) between September 2014 and December 2019 and offer new insights on factors that may be taken into account when optimizing preventive sanitary precautions and developing safe hospital environments. The collected data included variables such as gender, age, ethnicity, residency, CEB specimen type, co-detection of CEB with other multidrug-resistant bacteria, and length of hospital stay.

## Materials and Methods

### Data

We analyzed retrospective data on CEB isolates that could be retrieved from the laboratory and hospital information systems of our institute, which is part of the University Hospital of Bonn, Germany (UKB). The UKB is a tertiary referral and maximum care hospital with 1,300 beds. Every year about 50,000 inpatients and 35,000 emergencies are treated, and over 350,000 outpatient procedures are provided. The UKB serves mainly German residents but also attracts patients living outside of Germany, mainly in the Arabian Peninsula. Our microbiological diagnostic unit services the University Hospital Bonn and other hospitals in the area and receives an average of 178,000 clinical samples each year. All CEB isolates from September 2014 till December 2019 were traced in the laboratory information system using species and resistance keywords by one operator. Only first isolates were selected for each pathogen–patient combination.

The isolate information was complemented by accessible clinical patient information including gender, age, co-colonization by methicillin-resistant *Staphylococcus aureus* (MRSA) or vancomycin-resistant *Enterococcus* (VRE), date of hospital admission, hospitalization stay, oncological or intensive-care ward (ICU) stay, place of residency, and ethnicity. We constructed a database that was password protected and accessible by only three operators who ensured that all patient data were delinked from any results other than CEB and were fully de-identified prior to analysis after the establishment of residency and ethnicity. To infer patient ethnicity, we utilized the free version of the validated and data protection compliant software tool Onolytics (Version 2020, San Jose, California, US). Onolytics classifies names into 189 cultural ethnic and linguistics groupings. Where available, the results were further controlled for plausibility and accuracy using additional information such as patients’ nationality.

All data relevant to the study are included in the article or uploaded as **Supplementary Information**.

The ethics committee of the University Hospital Bonn confirmed that no ethics approval was required for this study.

### Screening and Surveillance Policy

Following German guidelines, multi-resistance of gram-negative rods (MRGNs) was defined on the basis of resistance of a pathogen against three (3MRGN) or four (4MRGN) of the following antibiotic groups: acylureidopenicillins, third and fourth generation cephalosporins, carbapenems, and fluoroquinolones. At our UKB and serviced hospitals primary MRGN screening is performed on all patients who had been hospitalized abroad within the past year, on all transfers without current MRGN screening as well as on all patients that were in the same room with a patient with a 4MRGN. Screening and surveillance body sites include the anal region, the inguinal region, the throat as well as wounds if present. Surveillance after admission is ward-dependent but generally performed at least weekly. Screening and surveillance samples for CEB were routinely cultured by the laboratory on selective ESBL-media, identified *via* MALDI-TOF MS (VITEK MS, Biomerieux, Marcy-l’Etoile, France) and susceptibility-tested with the VITEK 2 system (Biomerieux, Marcy-l’Etoile, France). Carbapenem-resistant *Enterobacterales*, *A. baumannii* and *P. aeruginosa* or isolates with an unusual carbapenem-susceptibility profile (ertapenem/imipenem/meropenem) are routinely genotyped in-institute for the presence of common resistance genes, using the Allplex Entero-DR (Seegene, Seoul, South Korea) and eazyplex SuperBug Acineto Assays (AmplexBiosystems, Giessen, Germany).

### Statistical Analysis

We used regression techniques to assess the association between age, sex, ethnicity, place of residency, and other parameters with the occurrence and/or type of CEB colonization in patients. This allowed construction of patient and/or hospitalization driven risk profiles. First, we report the average prevalence of different types of CEB in various patient populations. Then, using multivariate regressions, we test whether the uncovered differences between groups persist when we take into account the differentials in observable characteristics (*e.g.* age, sex, days of hospitalization, *etc.*) among individuals in these groups. To run the regressions we use the statistical software package Stata.

Our main results showing the relationship between demographic and hospitalization related characteristics with CEB colonization are shown in **Tables B1–B4 in Appendix B (Supplemental Material)**. Figures showing the absolute number and share of patients with a certain type of CEB colonization are shown throughout the text.

The p-values, marked with an asterisk (*) in these figures and throughout the text, refer to the significance (at the 0.05 or 0.01 level) of the point estimates obtained in the multivariate regression analysis (shown in **Tables B1–B4**).

To test the robustness of our results, we performed some additional sensitivity analyses. Results from univariate regression analysis (*i.e.* including only ethnicity or place of residence as independent variables and excluding all other covariates) are shown in **Tables B9–B12 in Appendix B**. Furthermore, due to the binary nature of the dependent variables (*e.g.* the prevalence of specific CEB in the patient), we also estimated the same specifications as in the multivariate analysis applying logistic models. The results of this application are shown in **Tables B13–B16 in Appendix B**. These sensitivity analyses show the same patterns of statistical significance and, hence, confirm our baseline results obtained with multivariate linear models.

### Clonality Analysis

We had access to the genomes of ten KPC-encoding *Enterobacter cloacae* complex isolates and MLST and OXA variant data of 12 OXA-48-encoding *K. pneumoniae* isolates from 2019 [see **Tables C1, C2 in Appendix C (Supplemental Material)**]. Genome analysis was performed with software tools (ResFinder, MLST, PlasmidFinder) of the CGE Server (Update June 8th 2020, Center for Genomic Epidemiology, DTU, Denmark).

## Results

### Detected CEB From 2014 to 2019

Between September 2014 and December 31st, 2019, 1,917 isolates from 1,384 patients were genotyped with the Allplex Entero-DR and eazyplex SuperBug Acineto Assays [see **Table A1 in Appendix A (Supplemental Material)**], and revealed 301 CEB isolates from UKB patients and 96 CEB isolates from patients of neighboring clinics. **Table 1** shows the demographic and clinical summary information for patients with detected CEB. Assuming that there is no gender imbalance regarding the overall amount of specimens received by our diagnostic unit, male patients (69.72%; 221/317) were more than twice as likely to be colonized with clinically relevant CEB compared to female patients (30.28%; 96/317). There were more CEB isolates during June, August, and October compared to the other months. However, clusters were mostly linked to small outbreaks rather than to seasonality.

**Table 1:** Summary demographics for patients with detected CEB isolates.

Demographic parameters	Patients n = 317 (%)	
**Age (years)**		
Mean (Min, Max)	56.95 (0, 97)	
**Sex**		
Female	96	(30.28%)
Male	221	(69.72%)
**Ethnicity**		
German	205	(64.67%)
Arabic	33	(10.41%)
Turkish	15	(4.73%)
Punjabi	13	(4.10%)
Somalian	11	(3.47%)
Kashmiri	4	(1.26%)
Other/Not available	36	(11.36%)
**Residency**		
Germany	209	(65.93%)
Arabian Peninsula	32	(10.09%)
Other/Not avaiable	76	(23.98%)
**Ward**		
ICU (but not oncological)	76	(23.98%)
Oncological (but not ICU)	40	(12.62%)
Oncological ICU	13	(4.10%)
Other	188	(59.31%)

### Species, Carbapenemases, and Specimens

Detected species and encoded carbapenemases are displayed in **Table 2**. Two hundred and fifty-three patients carried a single CEB species, and 64 patients carried multiple CEB species. Of these 64 patients, fifty-one, eleven, one, and one patients carried two, three, four and five different CEB, respectively, in 144 species in total. Among patients colonized by only one CEB, *P. aeruginosa* made up 26% (66/253) of all. Among patients that were detected to carry two or more CEB, *P. aeruginosa* constituted only 8% (11/142) (p <.01), and *Enterobacteriaceae* made up more. The OXA-48- and OXA-23-encoding *P. aeruginosa* and OXA-23-encoding *K. pneumoniae* isolates that appear in our statistic all occurred in 2014 and 2015 and were not cryopreserved in a sufficiently reliable traceable manner for their validity to now be confirmed in retrospect, given their rarity; DNA contaminations can, hence, not be excluded for these six isolates.

**Table 2:** Number and type of carbapenemases the most prevalent species encoded.

Species	Total	KPC	VIM	NDM	OXA-23	OXA-48	Others
*K. pneumoniae*	117	12	7	28	2	85	0
*Enterobacter cloacae* complex	46	11	21	6	0	7	1
*E. coli*	40	3	9	11	0	18	0
Other *Enterobacterales*	55	4	13	11	0	27	0
*P. aeruginosa*	74	0	63	5	1	3	4
*A. baumannii*	58	1	0	5	42	0	12
Total	390	31	113	66	45	140	17

The number of KPC, VIM, NDM, OXA-23, and OXA-48 like carbapenemases that had been detected as colonizers fluctuated each year between 2015 and 2019 (see **Figure A1 in Appendix A**). The majority of the KPC enzymes were detected in 2019 between June and August, due to an outbreak. The source of the outbreak had been timely traced, and no patients developed clinical CEB infections. The outbreak was mainly characterized by *Enterobacter cloacae* complex ST 419 [see **Table C1 in Appendix C (Supplemental Material)**]. In contrast, all KPC-encoding bacterial isolates detected between 2015 and 2018 were *K. pneumoniae* isolates and were detected only sporadically. Of all KPC-encoding isolates, 84% (26/31) were detected in screening and surveillance samples. Of VIM-encoding isolates, 31% (36/116) belonged to oncological and 35% (40/116) to ICU patients, making them the most frequently detected carbapenemases in both patient populations. The number of NDM carbapenemases grew steadily until 2018. ICU patients carried 26% (18/67) of the isolates, and oncological patients carried 29% (20/67). Numbers of OXA-23 carbapenemases showed only minimal variations, and only 22% (10/46) of the OXA-23-encoding bacterial strains belonged to female patients.

*K. pneumoniae* made up 61% (85/140) of OXA-48-encoding isolates. It is known that *bla*_OXA-48_ is on an IncL-type plasmid defective for the *tir* gene involved in the regulation of conjugation rendering this plasmid highly conjugative. Thus it has the ability to be transferred *in vivo* within different members of Enterobacterales. Genetic resistance profile and MLST-type of OXA-48-encoding *K. pneumoniae* isolates detected between January and October 2019 are displayed in **Table C2 in Appendix C (Supplemental Material)**. Of interest was that, every 10th patient colonized by an OXA-48-encoding isolate was colonized by at least two different OXA-48-encoding species, all belonging to the family of Enterobacteriaceae. Sixteen isolates, including 14 K*. pneumoniae* isolates, encoded OXA-48-like carbapenemases in addition to other carbapenemases. Fourteen percent (19/140) of the OXA-48-encoding isolates belonged to oncological patients and 24% (34/140) to ICU patients.

**Figure 1:**
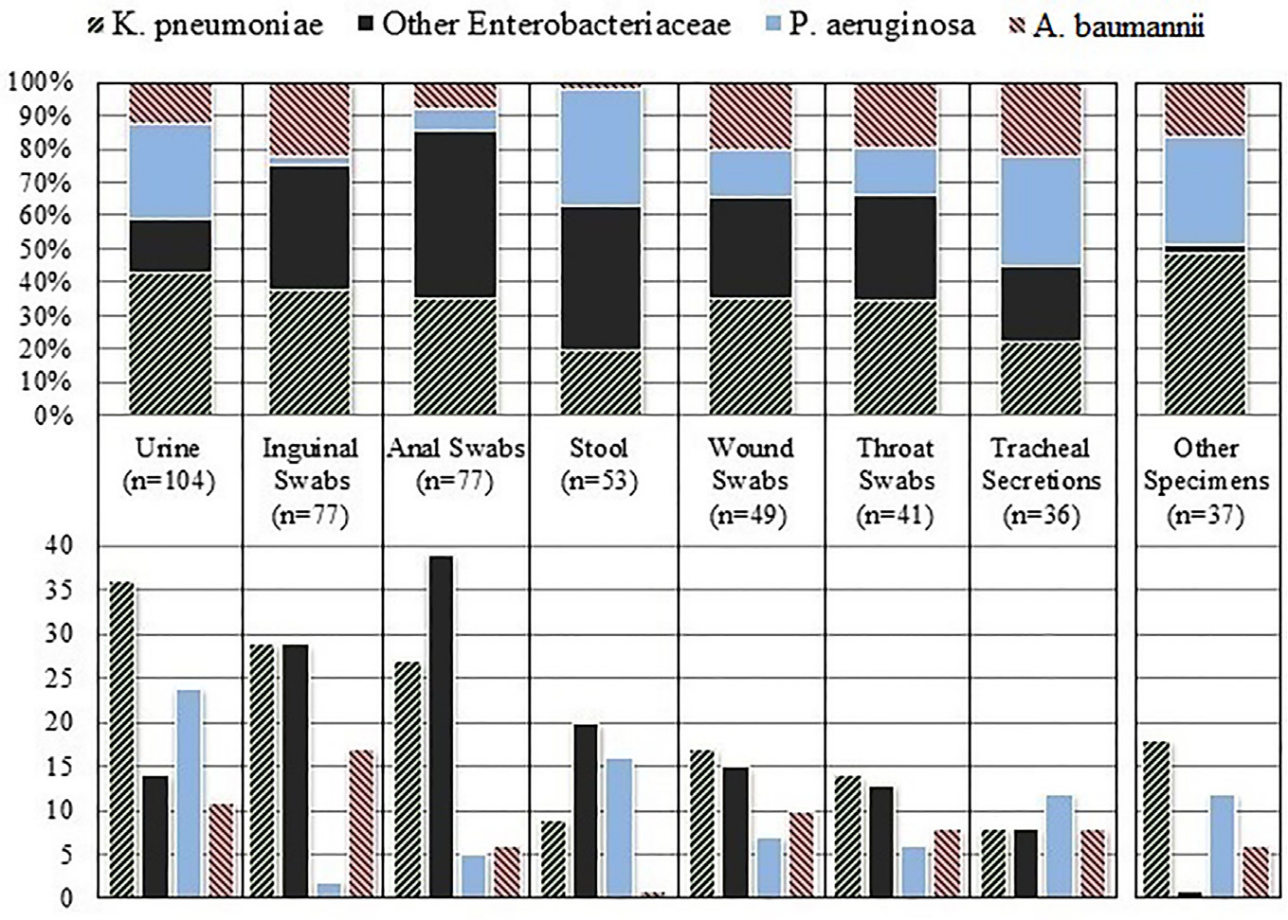
Relative (above) and absolute (below) quantities of carbapenemase-encoding species that were isolated from the respective type of clinical specimen.

**Figure 1** shows relative and absolute quantities of carbapenemase-encoding species that were isolated from the respective types of clinical specimen. Urine samples made up the majority (104) of all the specimen types in which CEB isolates were detected, followed by inguinal swabs (77), anal swabs (77), stool samples (53), wound swabs (49), throat swabs (41), tracheal secretions (36), and blood cultures (14). Nearly half (46%, 183/397) of all CEB were detected in screening and surveillance samples. The frequency with which CEB species were identified differed substantially by specimen type (see **Figure 1**). In our setting, inguinal swabs were the most efficient specimen for detecting carbapenemase-encoding *A. baumannii. P. aeruginosa* was more evenly found in the various specimen types, and the least efficient recovery was in anal and inguinal swabs.

### Age, Gender and Co-colonization With Other Resistant Bacteria

The total and relative frequencies with which certain carbapenemases and species were detected across different age groups are shown in **Figure A2 in Appendix A**. More than 83% (265/323) of the patients colonized by CEB were aged 40 or older, 51% (163/323) were 60 or older. No carbapenemase-encoding *P. aeruginosa* isolates were detected in patients below the age of 25 (n = 25). Among patients aged 25–29 years, including four oncological patients, a disproportionately high number (n=8/19) was found. While there are no gender differences in the average age, we found significant differences in the occurrence of CEB isolates in male and female patients. As the results of the multivariate regressions in **Tables B1–B4** show, VIM carbapenemases (p <.05*) and *Citrobacter* isolates (p <.05*) were significantly more common in female patients.

Thirty-four and sixty-seven patients were co-colonized with methicillin-resistant *Staphylococcus aureus* (MRSA) and vancomycin-resistant *Enterococcus* (VRE), respectively, and five of these patients with both. There were significantly less women co-colonized with MRSA, 5/34 (14.7%) (*vs* male, p = .04), compared to co-colonization with VRE, 22/67 (32.8%) (*vs* male p = .61).

### Role of Residency and Ethnicity

**Figure 2:**
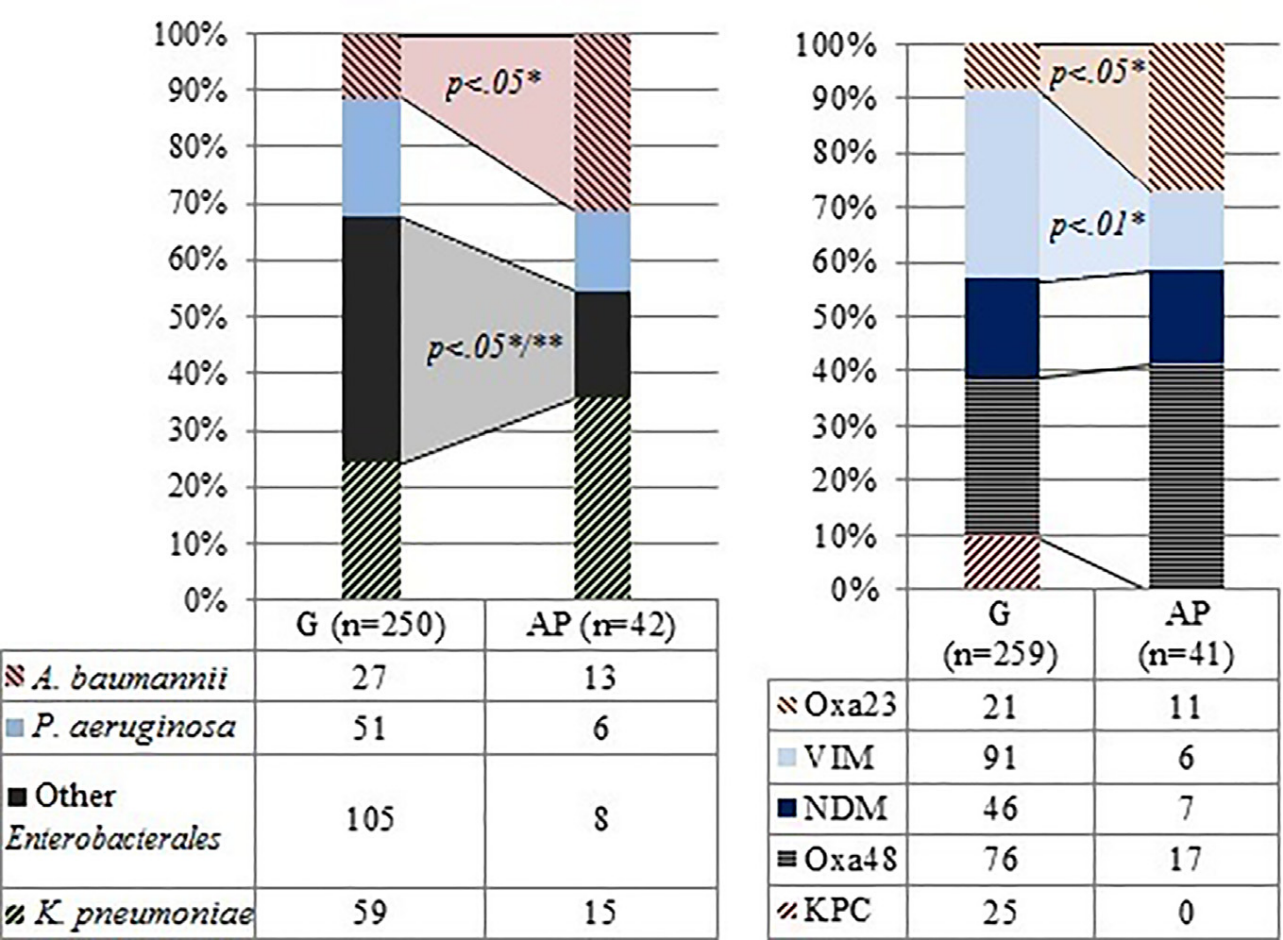
Species and carbapenemases patients resident in Germany (G) and on the Arabian Peninsula (AP) were detected with. *P-values refer to the significance of the point estimates in the regression analysis (see **Appendix B**). **P-value refers to Enterobacter isolates.

**Figure 3:**
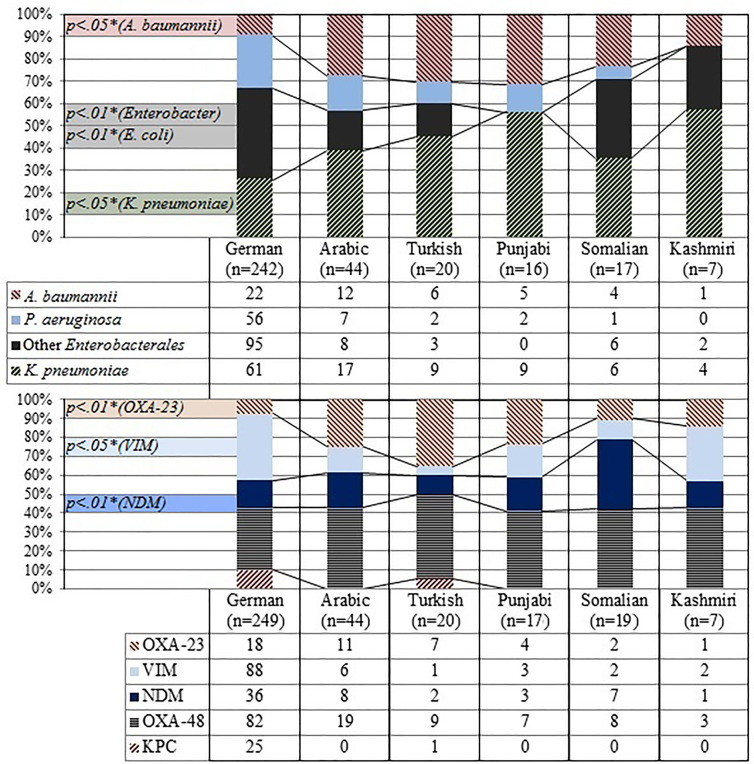
Summary of patient ethnicity and frequency of species and carbapenemases.*P-values refer to the significance of the point estimates in the regression analysis, baseline category for ethnicity is German (see **Appendix B**).

The human microbiome is very individual and has been correlated with factors such as ethnicity and geography (Brooks et al., 2018; Gaulke and Sharpton, 2018; Reinheimer et al., 2019). Hence, we investigated the presence of certain types of CEB in particular groups within our sample, identified by their ethnicity and country of residency. Two groups were considered for residence, patients residing in Germany (G-residents) and patients residing in the Arabian Peninsula (AP-residents) (see **Table 1**). AP-residents colonized with CEB were substantially younger (average age, 39 years) compared to G-residents (average age, 61 years) (t = 7.62, p <.01). Forty-four percent (14/32) of AP-residents were detected to carry CEB on admission, compared to only 23% (48/209) G-residents (p = .01). **Figure 2** shows the relative frequencies of species and carbapenemases among the two groups. The indicated p-values derive from the multivariate regressions shown in **Tables B1–B4**. *Enterobacter* isolates (p <.05*) and VIM enzymes (p <.01*) were more frequent among G-residents, while KPC enzymes were solely detected in patients in this group. In contrast, *A. baumannii* isolates (p <.05*) and OXA-23 carbapenemases (p <.05*) were more frequent among AP-residents. The ethnicity of patients had also been retrieved, as explained in the section *Materials and Methods* (see **Table 1**). **Figure 3** shows the relative frequencies of species and carbapenemases for each group. Again, p-values derive from the multivariate analysis. Although we report the uncovered differences among all ethnic groups in our sample, only German and Arabic ethnicities have a sufficiently high number of observations. Hence, results for the other groups should be read with caution. Controlling for all differential characteristics among groups, as age, sex, hospital stay *etc.*, patients of German ethnicity were less frequently colonized by *A. baumannii* than patients of Arabic ethnicity (p <.05*), and by *K. pneumoniae* than patients of Punjabi ethnicity (p <.05*). Conversely, they were more frequently colonized by *Enterobacter* isolates than patients of Arabic ethnicity (p <.01*). VIM enzymes were more frequently detected among patients of German ethnicity rather than among patients of Arabic (p <.05*), Somali (p <.05*), and Turkish ethnicity (p <.05*). On the other hand, patients of German ethnicity were less frequently colonized with bacteria harboring OXA-23 carbapenemases than patients of Arabic (p <.01*) and Turkish ethnicity (p <.01*) and by bacteria harboring NDM carbapenemases than patients of Somali ethnicity (p <.01*).

Our data suggests that residency and ethnicity play a role in terms of frequency with which certain carbapenemase-encoding species were detected in patients. Hence, we performed two further analyses. Firstly, we compared isolates of patients of German ethnicity residing in Germany with isolates of patients of Arabic, Somali, Turkish, Punjabi, or Kashmiri ethnicity residing in Germany (**Tables B5, B6 in Appendix B**). Secondly, we compared isolates of patients of Arabic ethnicity residing in Germany with isolates of patients of Arabic ethnicity residing on the Arabian Peninsula (**Tables B7, B8 in Appendix B**).

German ethnicity patients residing in Germany were less frequently colonized by OXA-23-encoding isolates compared to Turkish (p <.05*) and Arabic ethnicity patients residing in Germany (p <.05*), by OXA-48-encoding isolates compared to Somali ethnicity patients residing in Germany (p <.05*), and by VIM-encoding isolates compared to Kashmiri ethnicity patients. At the same time, they were associated more frequently with VIM enzymes than patients of Turkish ethnicity residing in Germany (p <.05*). Patients of Arabic ethnicity residing in Germany were more frequently colonized with carbapenemase-encoding *E. coli* isolates compared to patients of Arabic ethnicity residing in the Arabian Peninsula (p <.05*). Although the substantially smaller sample size challenges the consistency of these last results, they provide further suggestive evidence that confirms the significant role of ethnicity.

### Introduced vs. Hospital-Acquired

**Figure 4:**
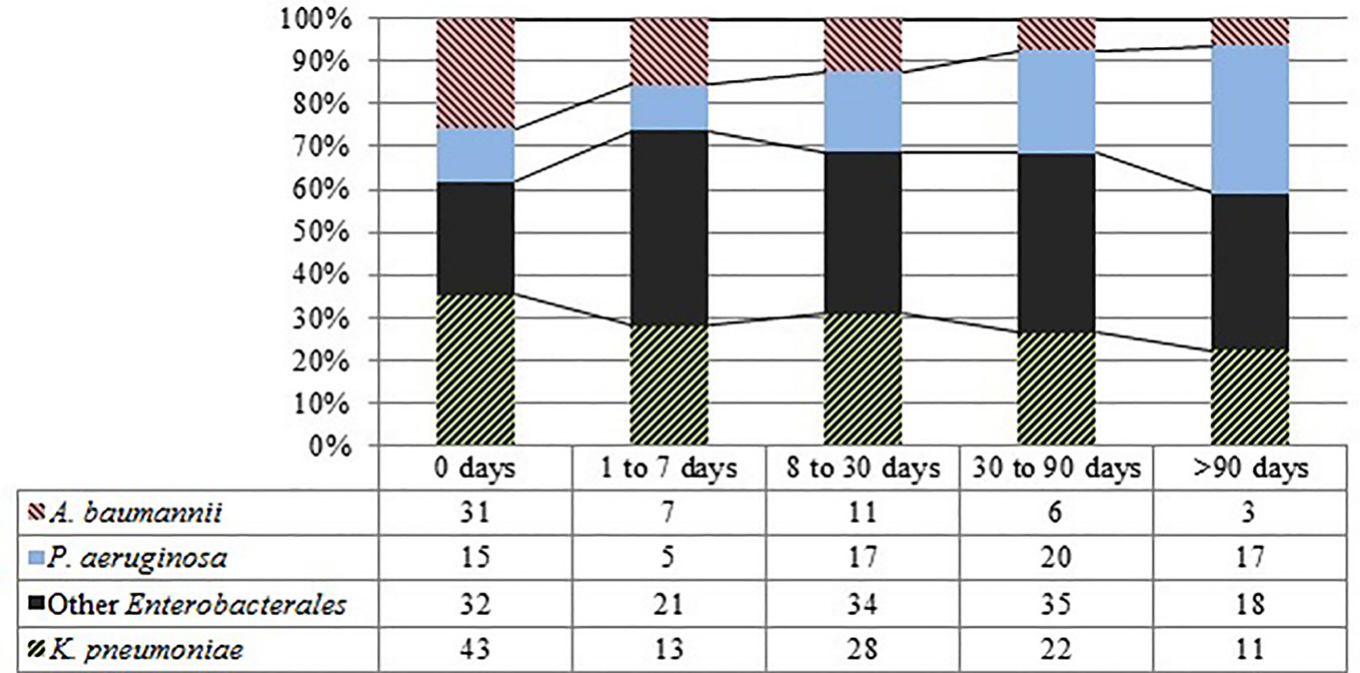
Total and relative amounts of species by LOS.

In order to differentiate CEB that are frequently introduced into the hospital from those that are more likely hospital-acquired, we studied CEB detection and the length of stay in the hospital (LOS). **Figure 4** demonstrates that carbapenemase-encoding *A. baumannii* isolates were more frequently detected in patients on hospital admission. Carbapenemase-encoding *P. aeruginosa* isolates on the other hand were more frequently detected with longer LOS.

In more than half of the cases *P. aeruginosa* was detected after more than four weeks LOS. The results of the multivariate analysis confirmed the existence of a statistically significant and negative relationship between the LOS and *P. aeruginosa*, *Proteus, Serratia, Klebsiella oxytoca*, and *Klebsiella aerogenes* as well as with production of OXA-48-like enzymes. *P. aeruginosa* was the most frequently isolated CEB from oncological and ICU patients. Especially oncological patients were colonized significantly more frequently by carbapenemase-encoding *P. aeruginosa* than by *A. baumannii* (p <.01).

## Discussion

As in other studies on multidrug-resistant gram-negative bacteria (GNB) (Kaase et al., 2016; Nicolas-Chanoine et al., 2019), in our study male patients were more prominently colonized with CEB. Methicillin-resistant *Staphylococcus aureus* (MRSA), but not vancomycin-resistant *Enterococcus* (VRE), more frequently colonized male patients (Humphreys et al., 2015; Markwart et al., 2019). The higher incidence of each, CEB and MRSA, in male patients explains the intensified gender imbalance in patients co-colonized by both. Higher MRSA-prevalence among men has been postulated to be linked to hand-hygiene and other behavioral factors as well as to immunological and endocrinological differences (Humphreys et al., 2015). Whether the same mechanisms apply for the higher occurrence of multidrug resistant gram-negative bacteria in males, too, remains uncertain.

The 2019 KPC outbreak highlights how explosively KPC-encoded resistance genes can spread in hospital settings despite surveillance and preventive measures. On the other hand, the quantity of carbapenemase-encoding bacteria (CEB) in screening and surveillance samples demonstrates the importance of screening and surveillance practices (Luebbert et al., 2013) to prevent and contain outbreaks. In our study OXA-48-like and VIM carbapenemases were the first and second most prevalent carbapenemases. However in 2018 NDM-carbapenemases were firstly detected more commonly than VIM-enzymes. The steadily growing number of NDM carbapenemases follows a countrywide trend (Robert-Koch-Institute, 2019). The interchangeable and compatible nature of OXA-48-like enzymes has been reported before (Pulss et al., 2018; Hamprecht et al., 2019). In our study these enzymes were encoded by many *Enterobacterales* and even by *P. aeruginosa. K. pneumoniae* confirmed its versatility in taking up resistance genes (Navon-Venezia et al., 2017). That *P. aeruginosa* is more frequently the only carbapenemase-encoding species carried by patients compared to *Enterobacteriaceae*, might be caused by the fact that the plasmid-subtypes generally carried by *P. aeruginosa* are less conjugative to other species, whereas plasmid-subtypes encoded by *Enterobacteriaceae* are more readily transferable to other *Enterobacteriaceae* (Pulss et al., 2018; Hamprecht et al., 2019; Sawa et al., 2020).

We hypothesize that several factors influence the different spectrum of CEB detected in anal swabs and stool specimen. In our experience, anal swabs are frequently erroneously collected by only swabbing the superficial area surrounding the anal sphincter. Also, only stool samples of oncological patients are routinely cultured on media on which CEB grow. Among the clinical specimens that we received, inguinal swabs were the best for detecting carbapenemase-encoding *A. baumannii*. The likelihood with which different CEB may be detected in various specimen types should be considered when screening patients in clinical practice.

The apparent decline in occurrence of CEB after the age of 70 observed in our study is solely due to the decreasing number of people in that age group. Colonization with multidrug-resistant bacteria is generally antibiotic- and healthcare associated (Logan and Weinstein, 2017; Nicolas-Chanoine et al., 2019; Segagni Lusignani et al., 2020) and hence more prevalent among the older population. Also, patients residing in foreign countries in need of medical attention coming to Germany for treatment likely more frequently have a long history of hospital stays and antimicrobial therapies. This also explains the higher amount of patients already colonized with CEB at hospital admission among AP-residents compared to G-residents, even though endemicity might as well play a role to a certain degree (van der Bij and Pitout, 2012). The age difference between the two groups may also be linked to the more than 15 years higher life expectancy in Germany compared to several countries on the Arabian Peninsula.

Our data suggests that besides the place of residence, ethnicity plays a role in terms of frequency with which certain CEB are detected in patients. Given the fact that the variability in the microbial species colonizing the human body has been shown to be linked to ethnicity as well as geography (Brooks et al., 2018; Gaulke and Sharpton, 2018; Reinheimer et al., 2019), we are not surprised by this result. Even though statistically significant in our study, confirmation by further studies is required due to the possibility of bias with smaller sample sizes. If confirmed, global distribution patterns of carbapenemases should be considered with these new insights. A higher likelihood of certain CEB in certain patient groups would offer the possibility to further tailor screening and diagnostic approaches as well as patient care, aiming for personalized and efficient precision medicine. On the other hand, there might currently be patients disadvantaged in healthcare settings because of their ethnicity, OXA-enzymes being the most challenging carbapenemases to detect with routine screening processes (Koroska et al., 2017). Culture media selective for carbapenem-resistant bacteria might for example be opportune for the above mentioned patient groups. The result is, however, challenged by the potentially different socioeconomic characteristics of patients. In particular, German residents of Arabic ethnicity in our sample are likely to be in a very different economic situation than residents of the Arabian Peninsula. Unfortunately, with the data at our disposal we are not able to control for the socioeconomic characteristics of patients, but the topic should be addressed carefully in future research.

*A. baumannii* has been documented to swiftly spread within hospital environments if effective preventive measures are inadequate (Teare et al., 2019). We therefore assume hospital-acquired cases in our study to be low due to effective screening, hygiene, and isolation measures (Luebbert et al., 2013). *P. aeruginosa* is documented to predominantly infect critically ill as well as immuno-suppressed patients (Aloush et al., 2006; Tsao et al., 2018) and its infection risk increases with longer LOS. Our study confirms these findings. A previous study showed that carbapenemase-encoding *P. aeruginosa* isolates detected in the siphons of the oncological ward mostly belong to the rare sequence type ST823 (Sib et al., 2019). Future studies should address if this and other detected sequence types match those detected in patients.

Given its frequent presence in hospital-wastewater, hotspots such as the drains, traps, sinks, faucets and toilets have undergone extensive remodeling. Nevertheless, our results and previous findings of the biofilms in the wastewater networks (Sib et al., 2019) further highlight the need to continuously rethink hospital-built environments for safety (Kizny Gordon et al., 2017; Hopman et al., 2019).

## Conclusions

We analyzed 397 carbapenemase-encoding isolates detected during a 5 year period with the associated demographic and clinical patient information. We found evidence of a role for ethnicity in the type of CEB colonization, and confirmed the important role of *P. aeruginosa* among complications of prolonged hospital stays. Continued research will further elucidate the observed gender differences in the frequency of MDR-GNB colonization/infection and the various microbial spectra linked to ethnicity and residency. The way global distribution patterns of carbapenemases are seen and studied may be substantially influenced and new screening, diagnosis, and patient care may be offered. These include more effective screening for challenging resistance enzymes linked with ethnicity/residence and the awareness of the optimal specimen for the various carbapenemase-encoding bacteria. Finally, our study has shown that even when extensive safety precautions are in place, not all hospital-acquired pathogens can be equally well contained, which prompts to continuously rethink hospital-built environments and further optimize all precautions according to risk factors and the spectrum of expected pathogens.

## Data Availability Statement

The original contributions presented in the study are included in the article/**Supplementary Material**. Further inquiries can be directed to the corresponding author.

## Author Contributions

CN and MP contributed to study design, data collection, data analysis, data interpretation, the literature search, and writing of the report. CB, GN, and IH contributed to data analysis, data interpretation, and writing of the report. GB contributed to the literature search, data interpretation, and writing of the report. AH contributed to data interpretation and writing of the report. All authors contributed to the article and approved the submitted version.

## Conflict of Interest

IH was employed by the company H.I.M.A. Consulting.

The remaining authors declare that the research was conducted in the absence of any commercial or financial relationships that could be construed as a potential conflict of interest.
