# Supplementary material for: Global Distribution Patterns of Carbapenemase-Encoding Bacteria in a New Light: Clues on a Role for Ethnicity
Source: Front Cell Infect Microbiol. 2021 Jun 29;11:659753. doi: 10.3389/fcimb.2021.659753 (PMC8276097; doi:10.3389/fcimb.2021.659753)
Supplement: Supplementary file 1 [file DataSheet_1.pdf]

Appendix A Additional Tables and Figures

Table A1: Table CEB

Year	Number of Genotyped Isolates	Number of Detected CEB
2014	96	21
2015	406	88
2016	325	79
2017	309	61
2018	349	56
2019	432	92
Total	1917	397

Figure A1: Number of detected carbapenemases each year between 2015 and 2019 highlighting the overall trend.

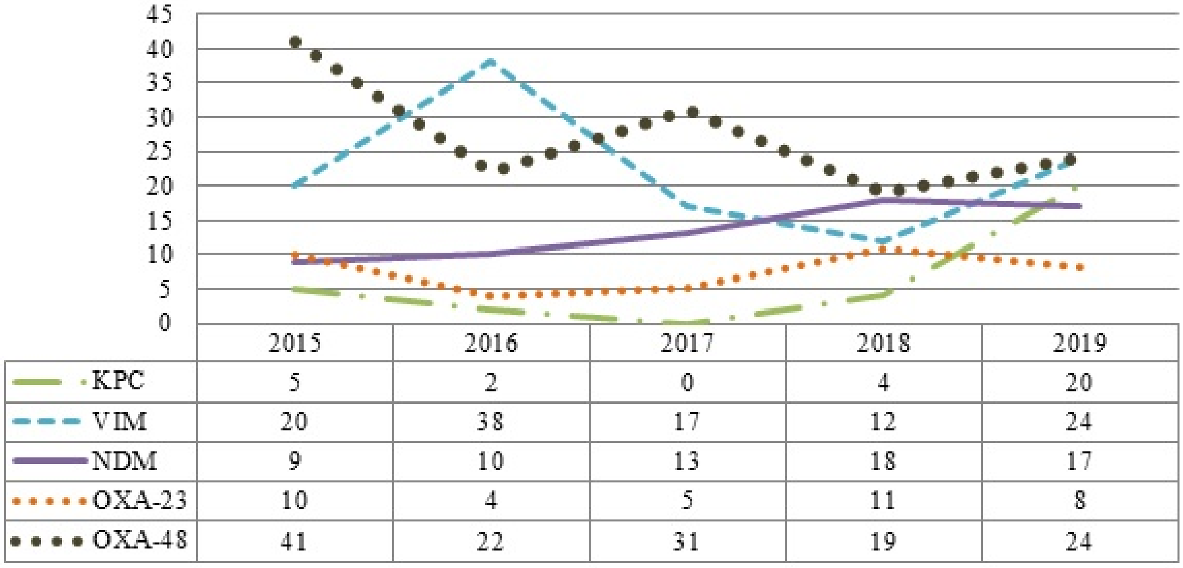

	2015	2016	2017	2018	2019
KPC	5	2	0	4	20
VIM	20	38	17	12	24
NDM	9	10	13	18	17
OXA-23	10	4	5	11	8
OXA-48	41	22	31	19	24

Figure A2: Relative (left) and absolute (right) quantities of the most clinically relevant species (above) and carbapenemases (below) detected among patients of different age groups.

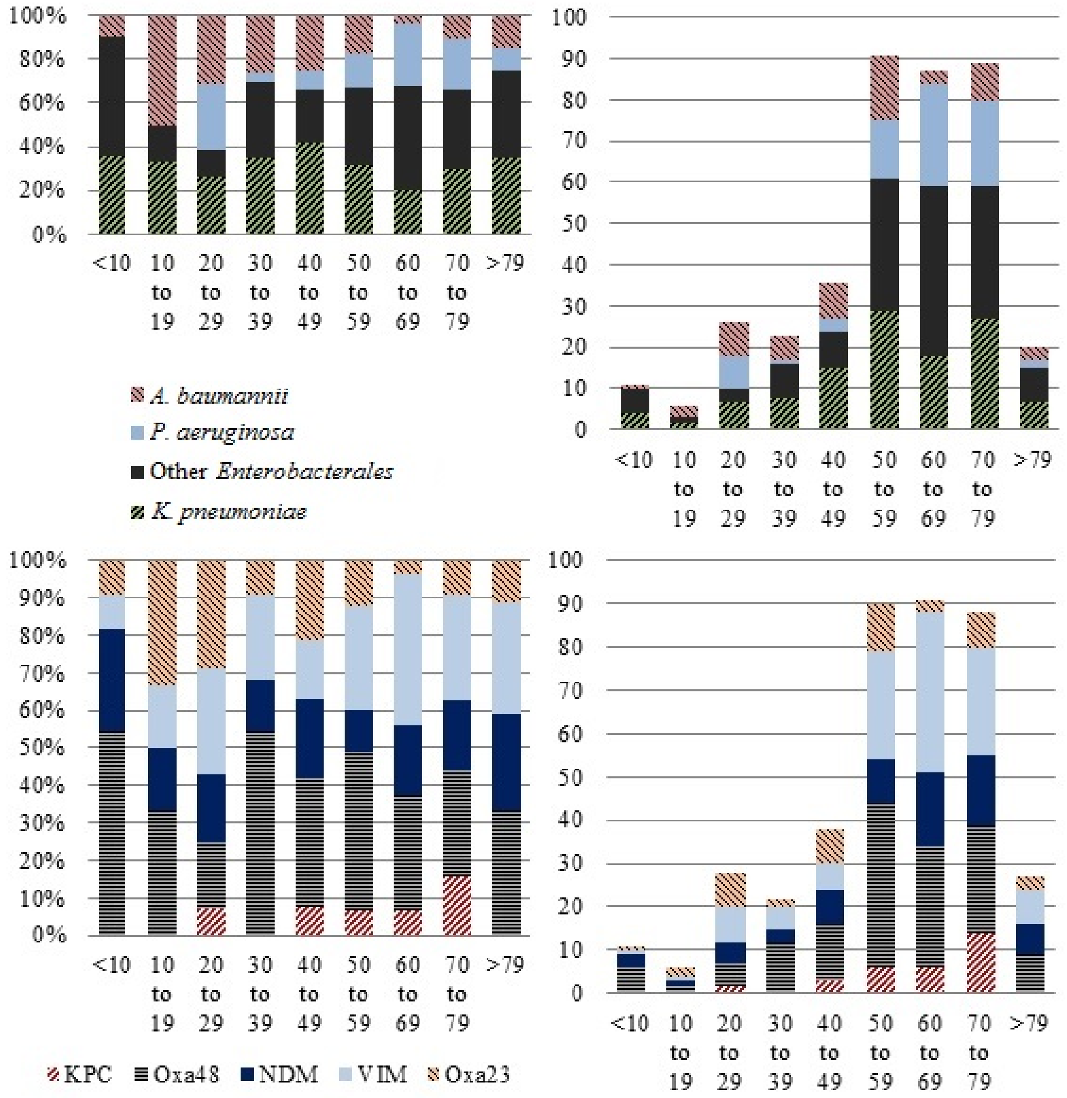

Appendix B Full Tables of Regression Analysis

B.1 Multivariate Linear Regressions

Table B1: Likelihood of colonization with carbapenemase-encoding Species for different Residency - linear regression analysis

	A.baumannii b/p-value/CI95	Citrobact b/p-value/CI95	E.coli b/p-value/CI95	Enterobact b/p-value/CI95	K.pneumoniae b/p-value/CI95	Other Enterobact b/p-value/CI95	Other Nonfermenter b/p-value/CI95	P.aearuginosa b/p-value/CI95
Residency								
Arabian Peninsula	0.136**	-0.027	-0.014	-0.122**	0.095	-0.056	-0.026	0.013
	0.0289	0.5423	0.8012	0.0354	0.2464	0.2548	0.2657	0.8423
	[0.014,0.258]	[-0.112,0.059]	[-0.120,0.093]	[-0.236,-0.008]	[-0.066,0.255]	[-0.152,0.040]	[-0.071,0.020]	[-0.119,0.146]
Other	-0.026	0.021	-0.068*	-0.049	0.122**	0.006	-0.028	0.022
	0.5759	0.5306	0.0981	0.2665	0.0469	0.8671	0.1044	0.6714
	[-0.118,0.066]	[-0.044,0.085]	[-0.148,0.013]	[-0.134,0.037]	[0.002,0.243]	[-0.066,0.078]	[-0.062,0.006]	[-0.078,0.121]
Control variables								
Age	-0.000	-0.000	0.000	0.002	0.001	-0.005	-0.003**	0.005
	0.9613	0.8793	0.8916	0.6454	0.7751	0.1325	0.0290	0.2572
	[-0.008,0.007]	[-0.006,0.005]	[-0.006,0.007]	[-0.005,0.009]	[-0.009,0.011]	[-0.011,0.001]	[-0.006,-0.000]	[-0.003,0.013]
Age × Age	-0.000	0.000	0.000	-0.000	-0.000	0.000	0.000***	-0.000
	0.4668	0.7497	0.8934	0.7263	0.6860	0.1732	0.0097	0.4641
	[-0.000,0.000]	[-0.000,0.000]	[-0.000,0.000]	[-0.000,0.000]	[-0.000,0.000]	[-0.000,0.000]	[0.000,0.000]	[-0.000,0.000]
Female	-0.024	-0.053**	0.056*	0.020	0.067	0.002	-0.009	-0.058
	0.5336	0.0495	0.0972	0.5832	0.1882	0.9417	0.5216	0.1641
	[-0.100,0.052]	[-0.106,-0.000]	[-0.010,0.122]	[-0.051,0.091]	[-0.033,0.166]	[-0.057,0.062]	[-0.037,0.019]	[-0.141,0.024]
Oncological:yes	-0.148***	0.033	0.013	-0.016	-0.116*	-0.014	0.071***	0.176***
	0.0031	0.3410	0.7600	0.7346	0.0749	0.7227	0.0001	0.0012
	[-0.245,-0.050]	[-0.035,0.102]	[-0.072,0.098]	[-0.107,0.075]	[-0.245,0.012]	[-0.091,0.063]	[0.035,0.108]	[0.070,0.282]
History of ICU:yes	0.026	0.083*	0.015	0.011	-0.061	-0.041	-0.046*	0.013
	0.7079	0.0917	0.8096	0.8657	0.5064	0.4541	0.0791	0.8603
	[-0.111,0.163]	[-0.013,0.179]	[-0.105,0.135]	[-0.117,0.139]	[-0.242,0.119]	[-0.149,0.067]	[-0.097,0.005]	[-0.136,0.162]
ICU:yes	-0.119*	-0.058	-0.017	0.072	0.046	0.082	0.013	-0.021
	0.0903	0.2413	0.7853	0.2707	0.6159	0.1370	0.6081	0.7839
	[-0.256,0.019]	[-0.154,0.039]	[-0.137,0.103]	[-0.056,0.201]	[-0.135,0.227]	[-0.026,0.190]	[-0.038,0.065]	[-0.170,0.128]
Days hospitalised	-0.001	-0.000	-0.000	-0.000	-0.000	0.001**	0.000	0.001***
	0.1017	0.3897	0.2087	0.6145	0.3989	0.0138	0.8415	0.0078
	[-0.001,0.000]	[-0.001,0.000]	[-0.001,0.000]	[-0.001,0.000]	[-0.001,0.001]	[0.000,0.001]	[-0.000,0.000]	[0.000,0.002]
Constant	0.332***	0.055	0.071	0.072	0.266*	0.166**	0.077**	-0.039
	0.0018	0.4603	0.4417	0.4687	0.0556	0.0462	0.0495	0.7354
	[0.124,0.539]	[-0.091,0.200]	[-0.110,0.252]	[-0.122,0.265]	[-0.006,0.539]	[0.003,0.329]	[0.000,0.155]	[-0.264,0.187]
Observations	390	390	390	390	390	390	390	390
R^2	0.096	0.022	0.026	0.036	0.045	0.029	0.095	0.080

Notes: Values of b show the estimated coefficients of linear probability models on the likelihood that the respective Species were detected, together with their p-value and confidence interval (95%). Baseline category for Residency is Germany. Statistical significance level * $p < 0.10$, ** $p < 0.05$, *** $p < 0.01$.

Table B2: Likelihood of colonization with carbapenemase-encoding Species for different Ethnicity - linear regression analysis

	A.baumannii b/p-value/CI95	Citrobact b/p-value/CI95	E.coli b/p-value/CI95	Enterobact b/p-value/CI95	K.pneumoniae b/p-value/CI95	Other Enterobact b/p-value/CI95	Other Nonfermenter b/p-value/CI95	P.aearuginosa b/p-value/CI95
Ethnicity								
Arabic	0.155**	-0.041	-0.002	-0.162***	0.105	-0.027	-0.023	-0.005
	0.0104	0.3285	0.9706	0.0040	0.1851	0.5689	0.3155	0.9393
	[0.037,0.274]	[-0.124,0.042]	[-0.106,0.102]	[-0.272,-0.052]	[-0.050,0.260]	[-0.120,0.066]	[-0.067,0.022]	[-0.134,0.124]
Kashmiri	-0.017	-0.073	-0.091	0.004	0.283	0.091	-0.008	-0.189
	0.8962	0.4332	0.4354	0.9764	0.1033	0.3823	0.8755	0.1878
	[-0.278,0.243]	[-0.255,0.110]	[-0.319,0.138]	[-0.238,0.246]	[-0.058,0.624]	[-0.114,0.296]	[-0.105,0.090]	[-0.472,0.093]
Punjabi	0.140	-0.078	-0.059	-0.131	0.292**	-0.072	-0.021	-0.070
	0.1313	0.2276	0.4666	0.1269	0.0163	0.3196	0.5400	0.4881
	[-0.042,0.322]	[-0.206,0.049]	[-0.218,0.100]	[-0.300,0.037]	[0.054,0.530]	[-0.215,0.070]	[-0.089,0.047]	[-0.267,0.127]
Somalian	0.103	-0.076	0.152*	-0.118	0.101	0.063	-0.050	-0.175*
	0.2497	0.2257	0.0518	0.1549	0.3855	0.3662	0.1314	0.0698
	[-0.072,0.277]	[-0.198,0.047]	[-0.001,0.305]	[-0.280,0.045]	[-0.128,0.330]	[-0.074,0.201]	[-0.116,0.015]	[-0.365,0.014]
Turkish	0.132	-0.086	-0.031	-0.137*	0.176	0.054	-0.024	-0.084
	0.1106	0.1367	0.6705	0.0738	0.1038	0.4046	0.4416	0.3475
	[-0.030,0.294]	[-0.200,0.027]	[-0.173,0.111]	[-0.288,0.013]	[-0.036,0.388]	[-0.073,0.181]	[-0.085,0.037]	[-0.259,0.092]
Other	0.025	0.030	0.005	0.034	-0.050	0.078*	-0.025	-0.097
	0.6489	0.4452	0.9169	0.5064	0.4887	0.0727	0.2306	0.1058
	[-0.083,0.133]	[-0.046,0.105]	[-0.090,0.100]	[-0.067,0.135]	[-0.192,0.092]	[-0.007,0.163]	[-0.065,0.016]	[-0.214,0.021]
Control variables								
Age	-0.000	-0.001	0.001	0.001	0.002	-0.004	-0.003**	0.004
	0.9403	0.8084	0.7950	0.6874	0.7094	0.1622	0.0260	0.3138
	[-0.008,0.007]	[-0.006,0.005]	[-0.006,0.007]	[-0.006,0.008]	[-0.008,0.012]	[-0.010,0.002]	[-0.006,-0.000]	[-0.004,0.012]
Age × Age	-0.000	0.000	0.000	-0.000	-0.000	0.000	0.000**	-0.000
	0.5484	0.7621	0.9394	0.7318	0.6741	0.1666	0.0102	0.4251
	[-0.000,0.000]	[-0.000,0.000]	[-0.000,0.000]	[-0.000,0.000]	[-0.000,0.000]	[-0.000,0.000]	[0.000,0.000]	[-0.000,0.000]
Female	-0.016	-0.062**	0.056	0.014	0.080	-0.001	-0.008	-0.062
	0.6821	0.0246	0.1034	0.7056	0.1166	0.9674	0.5671	0.1399
	[-0.092,0.061]	[-0.115,-0.008]	[-0.011,0.123]	[-0.057,0.085]	[-0.020,0.180]	[-0.061,0.059]	[-0.037,0.020]	[-0.145,0.021]
Oncological:yes	-0.133***	0.023	0.021	-0.008	-0.132**	-0.019	0.080***	0.168***
	0.0060	0.4965	0.6187	0.8666	0.0374	0.6088	0.0000	0.0014
	[-0.227,-0.038]	[-0.043,0.089]	[-0.062,0.104]	[-0.095,0.080]	[-0.255,-0.008]	[-0.094,0.055]	[0.044,0.115]	[0.065,0.270]
History of ICU:yes	0.054	0.066	0.044	0.013	-0.091	-0.048	-0.037	-0.002
	0.4199	0.1635	0.4497	0.8311	0.3001	0.3646	0.1464	0.9783
	[-0.078,0.186]	[-0.027,0.158]	[-0.071,0.160]	[-0.109,0.136]	[-0.264,0.082]	[-0.152,0.056]	[-0.086,0.013]	[-0.145,0.141]
ICU:yes	-0.122*	-0.058	-0.015	0.077	0.052	0.092*	0.010	-0.035
	0.0818	0.2382	0.8117	0.2384	0.5755	0.0945	0.7109	0.6417
	[-0.260,0.016]	[-0.155,0.039]	[-0.136,0.106]	[-0.051,0.205]	[-0.129,0.232]	[-0.016,0.201]	[-0.042,0.061]	[-0.185,0.114]
Days hospitalised	-0.001	-0.000	-0.000	-0.000	-0.000	0.001***	0.000	0.001***
	0.1405	0.3590	0.2085	0.4794	0.4441	0.0091	0.9681	0.0098
	[-0.001,0.000]	[-0.001,0.000]	[-0.001,0.000]	[-0.001,0.000]	[-0.001,0.001]	[0.000,0.001]	[-0.000,0.000]	[0.000,0.002]
Constant	0.274***	0.097	0.020	0.088	0.260*	0.128	0.077**	0.055
	0.0089	0.1842	0.8254	0.3638	0.0577	0.1192	0.0489	0.6245
	[0.069,0.479]	[-0.046,0.241]	[-0.159,0.200]	[-0.102,0.278]	[-0.009,0.528]	[-0.033,0.289]	[0.000,0.154]	[-0.167,0.278]
Observations	390	390	390	390	390	390	390	390
R^2	0.105	0.037	0.033	0.061	0.066	0.044	0.097	0.097

Notes: Values of b show the estimated coefficients of linear probability models on the likelihood that the respective Species were detected, together with their p-value and confidence interval (95%). Baseline category for Ethnicity is German. Statistical significance level * $p < 0.10$, ** $p < 0.05$, *** $p < 0.01$.

Table B3: Likelihood of Carbapenemases for different Residency - linear regression analysis

	GES<br>b/p-value/CI95	KPC<br>b/p-value/CI95	NDM<br>b/p-value/CI95	OXA-23<br>b/p-value/CI95	OXA-24-72<br>b/p-value/CI95	OXA-48<br>b/p-value/CI95	OXA-58<br>b/p-value/CI95	OXA-72<br>b/p-value/CI95	VIM<br>b/p-value/CI95
Residency									
Arabian Peninsula	0.047**	-0.072	-0.013	0.159***	-0.021	0.069	0.000	-0.017	-0.165**
	0.0498	0.1252	0.8445	0.0053	0.2379	0.4186	0.9830	0.3516	0.0352
	[0.000,0.094]	[-0.165,0.020]	[-0.148,0.121]	[0.048,0.271]	[-0.057,0.014]	[-0.098,0.236]	[-0.018,0.018]	[-0.053,0.019]	[-0.319,-0.012]
Other	-0.011	0.008	0.005	-0.021	0.013	0.069	0.009	-0.003	-0.094
	0.5437	0.8135	0.9295	0.6253	0.3517	0.2817	0.2178	0.8122	0.1114
	[-0.046,0.024]	[-0.061,0.078]	[-0.097,0.106]	[-0.105,0.063]	[-0.014,0.039]	[-0.057,0.195]	[-0.005,0.022]	[-0.030,0.024]	[-0.210,0.022]
Control variables									
Age	0.001	0.000	-0.004	-0.001	-0.000	0.001	0.000	0.001	0.003
	0.3234	0.9223	0.3422	0.7710	0.9484	0.9153	0.5152	0.4952	0.5793
	[-0.001,0.004]	[-0.005,0.006]	[-0.012,0.004]	[-0.008,0.006]	[-0.002,0.002]	[-0.010,0.011]	[-0.001,0.002]	[-0.001,0.003]	[-0.007,0.012]
Age × Age	-0.000	0.000	0.000	-0.000	-0.000	-0.000	-0.000	-0.000	-0.000
	0.6617	0.8855	0.2544	0.8578	0.5151	0.7452	0.4806	0.2827	0.8806
	[-0.000,0.000]	[-0.000,0.000]	[-0.000,0.000]	[-0.000,0.000]	[-0.000,0.000]	[-0.000,0.000]	[-0.000,0.000]	[-0.000,0.000]	[-0.000,0.000]
Female	0.010	-0.005	0.075*	-0.048	0.013	0.090*	-0.002	0.001	-0.120**
	0.4826	0.8638	0.0780	0.1768	0.2490	0.0906	0.6910	0.9598	0.0143
	[-0.019,0.040]	[-0.063,0.053]	[-0.008,0.159]	[-0.117,0.022]	[-0.009,0.035]	[-0.014,0.194]	[-0.014,0.009]	[-0.022,0.023]	[-0.215,-0.024]
Oncological:yes	0.014	-0.076**	0.140**	-0.109**	-0.012	-0.137**	-0.000	-0.013	0.269***
	0.4583	0.0440	0.0114	0.0174	0.4263	0.0446	0.9516	0.3731	0.0000
	[-0.023,0.052]	[-0.150,-0.002]	[0.032,0.247]	[-0.198,-0.019]	[-0.040,0.017]	[-0.271,-0.003]	[-0.015,0.014]	[-0.042,0.016]	[0.146,0.391]
History of ICU:yes	-0.023	0.203***	0.016	-0.008	-0.005	-0.231**	0.000	-0.013	0.012
	0.3927	0.0002	0.8378	0.8983	0.7992	0.0163	0.9774	0.5361	0.8913
	[-0.076,0.030]	[0.098,0.307]	[-0.136,0.168]	[-0.134,0.118]	[-0.045,0.035]	[-0.420,-0.043]	[-0.020,0.021]	[-0.053,0.028]	[-0.161,0.185]
ICU:yes	0.004	-0.091*	-0.037	-0.038	-0.008	0.115	-0.001	-0.005	0.083
	0.8966	0.0885	0.6317	0.5587	0.6855	0.2314	0.9304	0.8144	0.3483
	[-0.050,0.057]	[-0.195,0.014]	[-0.189,0.115]	[-0.164,0.089]	[-0.048,0.032]	[-0.074,0.304]	[-0.021,0.020]	[-0.045,0.036]	[-0.091,0.256]
Days hospitalised	-0.000	-0.000	-0.000	-0.000	-0.000	0.001*	-0.000	-0.000	0.000
	0.2104	0.9500	0.3338	0.2232	0.7965	0.0662	0.8445	0.5712	0.5725
	[-0.000,0.000]	[-0.001,0.001]	[-0.001,0.000]	[-0.001,0.000]	[-0.000,0.000]	[-0.000,0.002]	[-0.000,0.000]	[-0.000,0.000]	[-0.001,0.001]
Constant	-0.037	0.019	0.205*	0.250**	0.043	0.375**	-0.005	0.023	0.155
	0.3657	0.8144	0.0792	0.0101	0.1627	0.0100	0.7340	0.4615	0.2461
	[-0.117,0.043]	[-0.139,0.177]	[-0.024,0.435]	[0.060,0.440]	[-0.017,0.104]	[0.090,0.659]	[-0.036,0.025]	[-0.038,0.084]	[-0.107,0.416]
Observations	390	390	390	390	390	390	390	390	390
R^2	0.031	0.092	0.035	0.072	0.038	0.052	0.009	0.019	0.113

Notes: Values of b show the estimated coefficients of linear probability models on the likelihood that the respective carbapenemases were detected, together with their p-value and confidence interval (95%). Baseline category for Residency is Germany. Statistical significance level * $p < 0.10$, ** $p < 0.05$, *** $p < 0.01$.

Table B4: Likelihood of Carbapenemases for different Ethnicity - linear regression analysis

	GES<br>b/p-value/CI95	KPC<br>b/p-value/CI95	NDM<br>b/p-value/CI95	OXA-23<br>b/p-value/CI95	OXA-24-72<br>b/p-value/CI95	OXA-48<br>b/p-value/CI95	OXA-58<br>b/p-value/CI95	OXA-72<br>b/p-value/CI95	VIM<br>b/p-value/CI95
Ethnicity									
Arabic	0.009	-0.089*	0.057	0.160***	-0.015	0.032	-0.005	0.022	-0.180**
	0.7025	0.0543	0.3913	0.0038	0.3792	0.6973	0.5447	0.2016	0.0178
	[-0.037,0.055]	[-0.179,0.002]	[-0.074,0.187]	[0.052,0.268]	[-0.050,0.019]	[-0.131,0.195]	[-0.023,0.012]	[-0.012,0.057]	[-0.330,-0.031]
Kashmiri	-0.018	-0.104	0.035	0.030	-0.016	0.037	-0.007	-0.009	0.006
	0.7347	0.3017	0.8128	0.8058	0.6715	0.8391	0.7091	0.8131	0.9734
	[-0.119,0.084]	[-0.303,0.094]	[-0.252,0.321]	[-0.208,0.267]	[-0.092,0.060]	[-0.321,0.395]	[-0.046,0.032]	[-0.085,0.067]	[-0.322,0.333]
Punjabi	-0.010	-0.070	0.105	0.117	0.044	0.028	-0.009	-0.009	-0.116
	0.7853	0.3223	0.3041	0.1645	0.1056	0.8271	0.5327	0.7374	0.3192
	[-0.081,0.061]	[-0.209,0.069]	[-0.095,0.305]	[-0.048,0.283]	[-0.009,0.097]	[-0.222,0.278]	[-0.036,0.019]	[-0.062,0.044]	[-0.344,0.113]
Somalian	-0.023	-0.039	0.257***	0.021	0.043*	0.088	-0.007	-0.006	-0.232**
	0.5105	0.5652	0.0091	0.7995	0.0964	0.4703	0.6115	0.8203	0.0389
	[-0.091,0.045]	[-0.172,0.094]	[0.064,0.449]	[-0.139,0.180]	[-0.008,0.094]	[-0.152,0.329]	[-0.033,0.019]	[-0.057,0.045]	[-0.451,-0.012]
Turkish	-0.019	-0.027	-0.011	0.224***	-0.016	0.083	-0.008	-0.009	-0.267**
	0.5517	0.6652	0.9068	0.0030	0.5074	0.4614	0.5079	0.6941	0.0103
	[-0.082,0.044]	[-0.151,0.096]	[-0.189,0.168]	[0.076,0.372]	[-0.063,0.031]	[-0.139,0.306]	[-0.032,0.016]	[-0.057,0.038]	[-0.470,-0.063]
Other	0.002	0.014	0.104*	-0.040	0.005	-0.133*	-0.006	0.056***	-0.063
	0.9182	0.7345	0.0860	0.4282	0.7540	0.0807	0.4621	0.0005	0.3605
	[-0.040,0.045]	[-0.068,0.097]	[-0.015,0.224]	[-0.139,0.059]	[-0.027,0.037]	[-0.281,0.016]	[-0.022,0.010]	[0.024,0.087]	[-0.200,0.073]
Control variables									
Age	0.001	0.001	-0.003	-0.002	0.000	0.000	0.000	0.001	0.002
	0.5553	0.8537	0.5216	0.6336	0.7338	0.9389	0.5710	0.3629	0.6059
	[-0.002,0.004]	[-0.005,0.006]	[-0.011,0.006]	[-0.009,0.005]	[-0.002,0.003]	[-0.010,0.011]	[-0.001,0.001]	[-0.001,0.003]	[-0.007,0.012]
Age × Age	-0.000	0.000	0.000	0.000	-0.000	-0.000	-0.000	-0.000	-0.000
	0.8339	0.9800	0.2904	0.9796	0.3593	0.6980	0.4452	0.2883	0.8452
	[-0.000,0.000]	[-0.000,0.000]	[-0.000,0.000]	[-0.000,0.000]	[-0.000,0.000]	[-0.000,0.000]	[-0.000,0.000]	[-0.000,0.000]	[-0.000,0.000]
Female	0.010	-0.007	0.074*	-0.035	0.014	0.096*	-0.003	-0.003	-0.125**
	0.5306	0.8082	0.0866	0.3298	0.2180	0.0736	0.6046	0.7730	0.0109
	[-0.020,0.039]	[-0.066,0.051]	[-0.011,0.158]	[-0.104,0.035]	[-0.008,0.036]	[-0.009,0.201]	[-0.014,0.008]	[-0.026,0.019]	[-0.221,-0.029]
Oncological:yes	0.018	-0.085**	0.133**	-0.090**	-0.016	-0.157**	-0.003	-0.013	0.292***
	0.3406	0.0214	0.0122	0.0405	0.2518	0.0181	0.6320	0.3720	0.0000
	[-0.019,0.055]	[-0.157,-0.013]	[0.029,0.237]	[-0.176,-0.004]	[-0.044,0.011]	[-0.287,-0.027]	[-0.018,0.011]	[-0.040,0.015]	[0.174,0.411]
History of ICU:yes	-0.020	0.192***	0.028	0.016	-0.008	-0.255***	-0.004	-0.011	0.032
	0.4398	0.0002	0.7093	0.7998	0.6980	0.0060	0.6730	0.5765	0.7050
	[-0.072,0.031]	[0.091,0.292]	[-0.118,0.173]	[-0.105,0.136]	[-0.046,0.031]	[-0.436,-0.073]	[-0.024,0.015]	[-0.049,0.027]	[-0.134,0.198]
ICU:yes	0.004	-0.088	-0.031	-0.042	-0.007	0.119	-0.001	-0.007	0.072
	0.8818	0.1011	0.6858	0.5080	0.7214	0.2192	0.9451	0.7316	0.4119
	[-0.050,0.058]	[-0.193,0.017]	[-0.183,0.121]	[-0.168,0.083]	[-0.048,0.033]	[-0.071,0.308]	[-0.021,0.020]	[-0.047,0.033]	[-0.101,0.246]
Days hospitalised	-0.000	-0.000	-0.000	-0.000	-0.000	0.001*	-0.000	-0.000	0.000
	0.2092	0.9199	0.3455	0.3331	0.6905	0.0589	0.8282	0.6377	0.8228
	[-0.000,0.000]	[-0.001,0.001]	[-0.001,0.000]	[-0.001,0.000]	[-0.000,0.000]	[-0.000,0.002]	[-0.000,0.000]	[-0.000,0.000]	[-0.001,0.001]
Constant	-0.013	0.033	0.102	0.224**	0.029	0.434***	0.006	-0.002	0.186
	0.7467	0.6789	0.3761	0.0187	0.3408	0.0026	0.6971	0.9403	0.1575
	[-0.093,0.067]	[-0.123,0.189]	[-0.124,0.327]	[0.038,0.411]	[-0.031,0.089]	[0.152,0.715]	[-0.025,0.037]	[-0.062,0.057]	[-0.072,0.443]
Observations	390	390	390	390	390	390	390	390	390
R^2	0.021	0.100	0.059	0.096	0.050	0.061	0.008	0.055	0.130

Notes: Values of b show the estimated coefficients of linear probability models on the likelihood that the respective carbapenemases were detected, together with their p-value and confidence interval (95%). Baseline category for Ethnicity is German. Statistical significance level * $p < 0.10$, ** $p < 0.05$, *** $p < 0.01$.

Table B5: Likelihood of colonization with carbapenemase-encoding Species for different Ethnicity among German residents - linear regression analysis

	A.baumannii b/p-value/CI95	Citrobact b/p-value/CI95	E.coli b/p-value/CI95	Enterobact b/p-value/CI95	K.pneumoniae b/p-value/CI95	Other Enterobact b/p-value/CI95	Other Nonfermenter b/p-value/CI95	P.aearuginosa b/p-value/CI95
Ethnicity								
Arabic	0.087	-0.027	0.200*	-0.158	0.059	-0.054	-0.037	-0.070
	0.4216	0.7537	0.0823	0.2005	0.6900	0.5790	0.5118	0.5968
	[-0.125,0.299]	[-0.195,0.141]	[-0.026,0.426]	[-0.401,0.085]	[-0.232,0.349]	[-0.245,0.137]	[-0.147,0.073]	[-0.330,0.190]
Kashmiri	-0.169	0.016	-0.137	0.405	0.216	-0.104	0.040	-0.268
	0.4593	0.9294	0.5724	0.1211	0.4888	0.6137	0.7363	0.3398
	[-0.617,0.280]	[-0.340,0.372]	[-0.615,0.341]	[-0.108,0.919]	[-0.398,0.831]	[-0.509,0.301]	[-0.193,0.273]	[-0.819,0.284]
Punjabi	-0.231	-0.035	-0.091	-0.102	0.161	-0.030	-0.032	0.362
	0.3045	0.8428	0.7037	0.6906	0.6016	0.8821	0.7836	0.1916
	[-0.674,0.212]	[-0.387,0.316]	[-0.563,0.381]	[-0.609,0.404]	[-0.446,0.768]	[-0.430,0.370]	[-0.262,0.198]	[-0.182,0.906]
Somalian	0.092	-0.038	0.118	-0.095	0.200	0.071	-0.096*	-0.251*
	0.4044	0.6665	0.3149	0.4501	0.1848	0.4782	0.0927	0.0639
	[-0.125,0.308]	[-0.210,0.134]	[-0.113,0.349]	[-0.343,0.153]	[-0.096,0.497]	[-0.125,0.266]	[-0.209,0.016]	[-0.517,0.015]
Turkish	0.049	-0.043	-0.109	-0.136	0.145	0.182*	-0.030	-0.056
	0.6543	0.6183	0.3499	0.2786	0.3358	0.0673	0.5932	0.6754
	[-0.167,0.265]	[-0.215,0.128]	[-0.340,0.121]	[-0.384,0.111]	[-0.151,0.441]	[-0.013,0.377]	[-0.143,0.082]	[-0.322,0.209]
Other	0.003	0.130**	-0.057	0.058	-0.092	0.116**	-0.020	-0.137*
	0.9618	0.0111	0.4017	0.4247	0.2920	0.0463	0.5497	0.0811
	[-0.123,0.129]	[0.030,0.230]	[-0.191,0.077]	[-0.086,0.202]	[-0.265,0.080]	[0.002,0.229]	[-0.085,0.045]	[-0.292,0.017]
Control variables								
Age	-0.004	-0.000	0.006	0.002	-0.000	-0.003	-0.007**	0.006
	0.4733	0.9773	0.2714	0.7189	0.9595	0.5382	0.0154	0.3943
	[-0.015,0.007]	[-0.009,0.008]	[-0.005,0.018]	[-0.010,0.014]	[-0.015,0.014]	[-0.013,0.007]	[-0.012,-0.001]	[-0.007,0.019]
Age × Age	0.000	0.000	-0.000	0.000	-0.000	0.000	0.000***	-0.000
	0.8431	0.7694	0.3767	0.9578	0.7712	0.5352	0.0081	0.3725
	[-0.000,0.000]	[-0.000,0.000]	[-0.000,0.000]	[-0.000,0.000]	[-0.000,0.000]	[-0.000,0.000]	[0.000,0.000]	[-0.000,0.000]
Female	0.004	-0.080**	0.059	0.028	0.055	-0.016	-0.011	-0.040
	0.9255	0.0227	0.2088	0.5732	0.3594	0.6848	0.6419	0.4597
	[-0.082,0.090]	[-0.148,-0.011]	[-0.033,0.151]	[-0.070,0.127]	[-0.063,0.173]	[-0.094,0.062]	[-0.055,0.034]	[-0.146,0.066]
Oncological:yes	-0.119**	0.053	-0.015	-0.036	-0.113	-0.031	0.092***	0.169***
	0.0198	0.1925	0.7875	0.5355	0.1063	0.5029	0.0006	0.0072
	[-0.220,-0.019]	[-0.027,0.132]	[-0.121,0.092]	[-0.151,0.079]	[-0.250,0.024]	[-0.121,0.060]	[0.040,0.144]	[0.046,0.292]
History of ICU:yes	0.022	0.079	0.039	0.015	-0.050	-0.044	-0.056	-0.006
	0.7420	0.1400	0.5910	0.8431	0.5878	0.4728	0.1113	0.9468
	[-0.110,0.155]	[-0.026,0.184]	[-0.103,0.180]	[-0.136,0.167]	[-0.232,0.132]	[-0.164,0.076]	[-0.125,0.013]	[-0.168,0.157]
ICU:yes	-0.125*	-0.056	-0.018	0.088	-0.005	0.092	0.008	0.016
	0.0593	0.2830	0.7959	0.2475	0.9603	0.1230	0.8155	0.8406
	[-0.256,0.005]	[-0.160,0.047]	[-0.157,0.121]	[-0.061,0.237]	[-0.183,0.174]	[-0.025,0.210]	[-0.060,0.076]	[-0.144,0.176]
Days hospitalised	-0.000	-0.000	-0.001	-0.001**	-0.001	0.001**	0.000	0.001***
	0.6492	0.5267	0.2225	0.0459	0.3139	0.0154	0.7660	0.0039
	[-0.001,0.001]	[-0.001,0.000]	[-0.001,0.000]	[-0.002,-0.000]	[-0.002,0.001]	[0.000,0.002]	[-0.000,0.000]	[0.000,0.002]
Constant	0.373**	0.018	-0.081	0.004	0.393*	0.100	0.182**	0.010
	0.0130	0.8804	0.6128	0.9809	0.0552	0.4564	0.0199	0.9561
	[0.079,0.667]	[-0.215,0.251]	[-0.393,0.232]	[-0.332,0.340]	[-0.009,0.796]	[-0.165,0.366]	[0.029,0.334]	[-0.351,0.371]
Observations	243	243	243	243	243	243	243	243
R^2	0.083	0.072	0.049	0.081	0.064	0.064	0.126	0.124

Notes: Values of b show the estimated coefficients of linear probability models on the likelihood that the respective Species were detected, together with their p-value and confidence interval (95%). Baseline category for Ethnicity is German. Statistical significance level * $p < 0.10$, ** $p < 0.05$, *** $p < 0.01$.

Table B6: Likelihood of Carbapenemases for different Ethnicity among German residents - linear regression analysis

	GES<br>b/p-value/CI95	KPC<br>b/p-value/CI95	NDM<br>b/p-value/CI95	OXA-23<br>b/p-value/CI95	OXA-24-72<br>b/p-value/CI95	OXA-48<br>b/p-value/CI95	OXA-58<br>b/p-value/CI95	OXA-72<br>b/p-value/CI95	VIM<br>b/p-value/CI95
Ethnicity									
Arabic	-0.026	-0.069	-0.180	0.245**	-0.014	0.201	0.000	-0.011	-0.221
	0.5638	0.5000	0.1814	0.0114	0.5380	0.1977	.	0.7219	0.1687
	[-0.115,0.063]	[-0.268,0.131]	[-0.445,0.085]	[0.056,0.434]	[-0.058,0.030]	[-0.106,0.507]	[0.000,0.000]	[-0.072,0.050]	[-0.536,0.094]
Kashmiri	0.006	-0.175	-0.124	-0.081	-0.023	-0.429	0.000	-0.007	0.814**
	0.9468	0.4147	0.6621	0.6918	0.6217	0.1935	.	0.9146	0.0170
	[-0.181,0.194]	[-0.598,0.248]	[-0.684,0.436]	[-0.480,0.319]	[-0.117,0.070]	[-1.078,0.219]	[0.000,0.000]	[-0.136,0.122]	[0.147,1.481]
Punjabi	-0.027	-0.029	-0.174	-0.149	-0.023	0.158	0.000	-0.026	0.185
	0.7731	0.8898	0.5346	0.4587	0.6173	0.6270	.	0.6905	0.5802
	[-0.212,0.158]	[-0.447,0.388]	[-0.727,0.378]	[-0.543,0.246]	[-0.116,0.069]	[-0.482,0.798]	[0.000,0.000]	[-0.153,0.101]	[-0.473,0.843]
Somalian	-0.031	-0.008	0.195	0.033	-0.017	0.344**	0.000	-0.013	-0.274*
	0.5061	0.9390	0.1558	0.7341	0.4481	0.0313	.	0.6896	0.0946
	[-0.121,0.060]	[-0.212,0.196]	[-0.075,0.466]	[-0.160,0.226]	[-0.063,0.028]	[0.031,0.657]	[0.000,0.000]	[-0.075,0.050]	[-0.596,0.048]
Turkish	-0.028	-0.060	0.051	0.217**	-0.017	0.129	0.000	-0.016	-0.361**
	0.5470	0.5633	0.7080	0.0276	0.4648	0.4154	.	0.6134	0.0276
	[-0.118,0.063]	[-0.264,0.144]	[-0.218,0.321]	[0.024,0.409]	[-0.062,0.028]	[-0.183,0.442]	[0.000,0.000]	[-0.078,0.046]	[-0.683,-0.040]
Other	-0.021	-0.003	0.077	-0.055	-0.013	-0.068	0.000	0.062***	-0.049
	0.4263	0.9614	0.3339	0.3363	0.3331	0.4600	.	0.0009	0.6091
	[-0.074,0.031]	[-0.122,0.116]	[-0.080,0.234]	[-0.167,0.057]	[-0.039,0.013]	[-0.250,0.114]	[0.000,0.000]	[0.026,0.098]	[-0.236,0.138]
Control variables									
Age	0.001	0.001	-0.001	-0.002	-0.001	0.007	0.000	-0.001	-0.001
	0.5460	0.8246	0.9011	0.6513	0.5956	0.3696	.	0.6048	0.8973
	[-0.003,0.006]	[-0.009,0.011]	[-0.014,0.012]	[-0.012,0.007]	[-0.003,0.002]	[-0.008,0.022]	[0.000,0.000]	[-0.004,0.002]	[-0.017,0.015]
Age × Age	-0.000	0.000	0.000	0.000	0.000	-0.000	0.000	0.000	0.000
	0.6167	0.9397	0.8466	0.8405	0.9767	0.2553	.	0.8472	0.7170
	[-0.000,0.000]	[-0.000,0.000]	[-0.000,0.000]	[-0.000,0.000]	[-0.000,0.000]	[-0.000,0.000]	[0.000,0.000]	[-0.000,0.000]	[-0.000,0.000]
Female	-0.004	-0.013	0.005	-0.031	0.013	0.143**	0.000	0.006	-0.148**
	0.8248	0.7453	0.9328	0.4275	0.1498	0.0246	.	0.6593	0.0234
	[-0.040,0.032]	[-0.095,0.068]	[-0.103,0.112]	[-0.108,0.046]	[-0.005,0.031]	[0.018,0.268]	[0.000,0.000]	[-0.019,0.030]	[-0.277,-0.020]
Oncological:yes	0.024	-0.086*	0.115*	-0.067	-0.009	-0.121	0.000	-0.014	0.242***
	0.2653	0.0745	0.0707	0.1414	0.3891	0.1012	.	0.3485	0.0016
	[-0.018,0.066]	[-0.180,0.009]	[-0.010,0.240]	[-0.156,0.022]	[-0.030,0.012]	[-0.266,0.024]	[0.000,0.000]	[-0.043,0.015]	[0.093,0.391]
History of ICU:yes	-0.019	0.227***	-0.032	0.009	-0.009	-0.184*	0.000	-0.019	-0.032
	0.4935	0.0004	0.7035	0.8822	0.5357	0.0596	.	0.3328	0.7468
	[-0.075,0.036]	[0.102,0.352]	[-0.198,0.134]	[-0.109,0.127]	[-0.036,0.019]	[-0.376,0.008]	[0.000,0.000]	[-0.057,0.019]	[-0.229,0.165]
ICU:yes	0.005	-0.080	-0.058	-0.058	-0.004	0.063	0.000	-0.003	0.148
	0.8428	0.2004	0.4820	0.3233	0.7471	0.5112	.	0.8741	0.1342
	[-0.049,0.060]	[-0.203,0.043]	[-0.221,0.104]	[-0.174,0.058]	[-0.032,0.023]	[-0.125,0.251]	[0.000,0.000]	[-0.040,0.034]	[-0.046,0.341]
Days hospitalised	-0.000	-0.000	0.000	-0.000	-0.000	0.001	0.000	-0.000	-0.000
	0.3775	0.4740	0.8716	0.7356	0.8826	0.1985	.	0.8359	0.7745
	[-0.000,0.000]	[-0.001,0.000]	[-0.001,0.001]	[-0.001,0.001]	[-0.000,0.000]	[-0.000,0.002]	[0.000,0.000]	[-0.000,0.000]	[-0.001,0.001]
Constant	-0.009	-0.024	0.186	0.222*	0.048	0.211	0.000	0.054	0.306
	0.8831	0.8618	0.3190	0.0965	0.1260	0.3288	.	0.2106	0.1691
	[-0.132,0.114]	[-0.301,0.252]	[-0.181,0.552]	[-0.040,0.483]	[-0.014,0.109]	[-0.214,0.635]	[0.000,0.000]	[-0.031,0.138]	[-0.131,0.742]
Observations	243	243	243	243	243	243	243	243	243
R^2	0.022	0.127	0.064	0.088	0.039	0.099	.	0.084	0.134

Notes: Values of b show the estimated coefficients of linear probability models on the likelihood that the respective Species were detected, together with their p-value and confidence interval (95%). Baseline category for Ethnicity is German. Statistical significance level * $p < 0.10$, ** $p < 0.05$, *** $p < 0.01$.

Table B7: Likelihood of colonization with carbapenemase-encoding Species for different Residency among patients of Arabic Ethnicity - linear regression analysis

	A.baumannii<br>b/p-value/CI95	Citrobact<br>b/p-value/CI95	E.coli<br>b/p-value/CI95	Enterobact<br>b/p-value/CI95	K.pneumoniae<br>b/p-value/CI95	Other Enterobact<br>b/p-value/CI95	Other Nonfermenter<br>b/p-value/CI95	P.aearuginosa<br>b/p-value/CI95
Residency								
Arabian Peninsula	0.220	-0.005	-0.319**	0.000	-0.138	0.041	0.000	0.202
	0.2973	0.9366	0.0129	.	0.5361	0.6262	.	0.1969
	[-0.203,0.642]	[-0.145,0.134]	[-0.567,-0.072]	[0.000,0.000]	[-0.586,0.311]	[-0.128,0.209]	[0.000,0.000]	[-0.110,0.515]
Other	0.026	0.020	-0.390***	0.000	-0.108	0.078	0.000	0.374**
	0.9150	0.8079	0.0096	.	0.6779	0.4261	.	0.0444
	[-0.466,0.518]	[-0.143,0.182]	[-0.678,-0.101]	[0.000,0.000]	[-0.630,0.415]	[-0.118,0.274]	[0.000,0.000]	[0.010,0.738]
Control variables								
Age	0.004	-0.011**	0.013*	0.000	0.017	-0.014***	0.000	-0.009
	0.7548	0.0131	0.0840	.	0.2281	0.0089	.	0.3586
	[-0.022,0.030]	[-0.020,-0.002]	[-0.002,0.029]	[0.000,0.000]	[-0.011,0.044]	[-0.025,-0.004]	[0.000,0.000]	[-0.028,0.010]
Age × Age	-0.000	0.000**	-0.000**	0.000	-0.000	0.000**	0.000	0.000
	0.8313	0.0292	0.0402	.	0.2974	0.0280	.	0.2216
	[-0.000,0.000]	[0.000,0.000]	[-0.000,-0.000]	[0.000,0.000]	[-0.000,0.000]	[0.000,0.000]	[0.000,0.000]	[-0.000,0.000]
Female	-0.192	0.008	0.146	0.000	-0.082	-0.049	0.000	0.169
	0.2410	0.8828	0.1310	.	0.6341	0.4491	.	0.1634
	[-0.519,0.135]	[-0.100,0.116]	[-0.046,0.337]	[0.000,0.000]	[-0.429,0.265]	[-0.179,0.081]	[0.000,0.000]	[-0.072,0.411]
Oncological:yes	-0.405	-0.005	-0.215	0.000	0.501*	-0.061	0.000	0.184
	0.1268	0.9557	0.1650	.	0.0769	0.5590	.	0.3421
	[-0.930,0.121]	[-0.178,0.169]	[-0.522,0.093]	[0.000,0.000]	[-0.057,1.059]	[-0.270,0.149]	[0.000,0.000]	[-0.204,0.573]
History of ICU:yes	-0.185	0.016	0.013	0.000	0.551***	-0.040	0.000	-0.356**
	0.3131	0.7867	0.9056	.	0.0069	0.5823	.	0.0118
	[-0.552,0.182]	[-0.105,0.137]	[-0.202,0.227]	[0.000,0.000]	[0.162,0.941]	[-0.186,0.106]	[0.000,0.000]	[-0.627,-0.084]
ICU:yes	0.000	0.000	0.000	0.000	0.000	0.000	0.000	0.000
	.	.	.	.	.	.	.	.
	[0.000,0.000]	[0.000,0.000]	[0.000,0.000]	[0.000,0.000]	[0.000,0.000]	[0.000,0.000]	[0.000,0.000]	[0.000,0.000]
Days hospitalised	0.000	0.000	0.001	0.000	-0.005**	0.003***	0.000	0.001
	0.9925	0.8851	0.6700	.	0.0289	0.0006	.	0.4162
	[-0.004,0.004]	[-0.001,0.002]	[-0.002,0.003]	[0.000,0.000]	[-0.010,-0.001]	[0.001,0.005]	[0.000,0.000]	[-0.002,0.004]
Constant	0.266	0.247*	0.158	0.000	-0.059	0.306**	0.000	0.083
	0.4816	0.0532	0.4769	.	0.8819	0.0484	.	0.7669
	[-0.495,1.028]	[-0.004,0.498]	[-0.288,0.603]	[0.000,0.000]	[-0.868,0.749]	[0.002,0.609]	[0.000,0.000]	[-0.480,0.646]
Observations	42	42	42	42	42	42	42	42
R^2	0.212	0.248	0.361	.	0.232	0.437	.	0.367

Notes: Values of b show the estimated coefficients of linear probability models on the likelihood that the respective Species were detected, together with their p-value and confidence interval (95%). Baseline category for Ethnicity is German. Statistical significance level * $p < 0.10$, ** $p < 0.05$, *** $p < 0.01$.

Table B8: Likelihood of Carbapenemases for different Residency among patients of Arabic Ethnicity - linear regression analysis

	GES<br>b/p-value/CI95	KPC<br>b/p-value/CI95	NDM<br>b/p-value/CI95	OXA-23<br>b/p-value/CI95	OXA-24-72<br>b/p-value/CI95	OXA-48<br>b/p-value/CI95	OXA-58<br>b/p-value/CI95	OXA-72<br>b/p-value/CI95	VIM<br>b/p-value/CI95
Residency									
Arabian Peninsula	0.118	0.000	0.324*	-0.075	0.000	-0.402*	0.000	0.004	0.135
	0.1086	.	0.0526	0.7174	.	0.0669	.	0.9602	0.3859
	[-0.028,0.264]	[0.000,0.000]	[-0.004,0.653]	[-0.491,0.342]	[0.000,0.000]	[-0.834,0.030]	[0.000,0.000]	[-0.144,0.151]	[-0.177,0.447]
Other	0.038	0.000	0.387**	-0.330	0.000	-0.443*	0.000	0.147*	0.183
	0.6480	.	0.0473	0.1762	.	0.0824	.	0.0921	0.3124
	[-0.131,0.208]	[0.000,0.000]	[0.005,0.770]	[-0.815,0.156]	[0.000,0.000]	[-0.947,0.060]	[0.000,0.000]	[-0.025,0.318]	[-0.180,0.547]
Control variables									
Age	-0.003	0.000	0.025**	0.011	0.000	-0.017	0.000	-0.000	-0.008
	0.5366	.	0.0183	0.4011	.	0.2165	.	0.9452	0.4107
	[-0.012,0.006]	[0.000,0.000]	[0.004,0.045]	[-0.015,0.036]	[0.000,0.000]	[-0.043,0.010]	[0.000,0.000]	[-0.009,0.009]	[-0.027,0.011]
Age × Age	0.000	0.000	-0.000*	-0.000	0.000	0.000	0.000	-0.000	0.000
	0.2324	.	0.0513	0.3464	.	0.3875	.	0.9083	0.3440
	[-0.000,0.000]	[0.000,0.000]	[-0.000,0.000]	[-0.000,0.000]	[0.000,0.000]	[-0.000,0.000]	[0.000,0.000]	[-0.000,0.000]	[-0.000,0.000]
Female	-0.005	0.000	0.347***	-0.221	0.000	-0.001	0.000	-0.030	0.065
	0.9285	.	0.0090	0.1715	.	0.9929	.	0.6005	0.5849
	[-0.118,0.108]	[0.000,0.000]	[0.093,0.600]	[-0.543,0.101]	[0.000,0.000]	[-0.336,0.333]	[0.000,0.000]	[-0.144,0.084]	[-0.176,0.307]
Oncological:yes	-0.051	0.000	-0.307	-0.274	0.000	0.306	0.000	-0.029	0.231
	0.5717	.	0.1356	0.2892	.	0.2543	.	0.7514	0.2343
	[-0.232,0.130]	[0.000,0.000]	[-0.715,0.101]	[-0.792,0.244]	[0.000,0.000]	[-0.231,0.844]	[0.000,0.000]	[-0.212,0.155]	[-0.157,0.619]
History of ICU:yes	-0.051	0.000	0.095	0.040	0.000	0.345*	0.000	-0.060	-0.295**
	0.4214	.	0.5014	0.8232	.	0.0705	.	0.3515	0.0340
	[-0.177,0.076]	[0.000,0.000]	[-0.190,0.380]	[-0.322,0.402]	[0.000,0.000]	[-0.031,0.720]	[0.000,0.000]	[-0.188,0.069]	[-0.566,-0.024]
ICU:yes	0.000	0.000	0.000	0.000	0.000	0.000	0.000	0.000	0.000
	.	.	.	.	.	.	.	.	.
	[0.000,0.000]	[0.000,0.000]	[0.000,0.000]	[0.000,0.000]	[0.000,0.000]	[0.000,0.000]	[0.000,0.000]	[0.000,0.000]	[0.000,0.000]
Days hospitalised	0.000	0.000	-0.000	-0.001	0.000	0.000	0.000	0.000	0.001
	0.8431	.	0.7703	0.6085	.	0.9567	.	0.6980	0.5155
	[-0.001,0.002]	[0.000,0.000]	[-0.004,0.003]	[-0.005,0.003]	[0.000,0.000]	[-0.004,0.005]	[0.000,0.000]	[-0.001,0.002]	[-0.002,0.004]
Constant	-0.051	0.000	-0.760**	0.353	0.000	0.951**	0.000	0.063	0.179
	0.6953	.	0.0134	0.3455	.	0.0182	.	0.6314	0.5220
	[-0.313,0.211]	[0.000,0.000]	[-1.351,-0.168]	[-0.397,1.103]	[0.000,0.000]	[0.173,1.730]	[0.000,0.000]	[-0.202,0.329]	[-0.383,0.741]
Observations	42	42	42	42	42	42	42	42	42
R^2	0.179	.	0.371	0.192	.	0.302	.	0.157	0.284

Notes: Values of b show the estimated coefficients of linear probability models on the likelihood that the respective Species were detected, together with their p-value and confidence interval (95%). Baseline category for Ethnicity is German. Statistical significance level * $p < 0.10$, ** $p < 0.05$, *** $p < 0.01$.

B.2 Univariate Linear Regressions

Table B9: Likelihood of colonization with carbapenemase-encoding Species for different Residency - linear regression analysis

	A.baumannii b/p-value/CI95	Citrobact b/p-value/CI95	E.coli b/p-value/CI95	Enterobact b/p-value/CI95	K.pneumoniae b/p-value/CI95	Other Enterobact b/p-value/CI95	Other Nonfermenter b/p-value/CI95	P.aearuginosa b/p-value/CI95
Residency								
Arabian Peninsula	0.202***	-0.040	-0.029	-0.124**	0.117	-0.036	-0.028	-0.061
	0.0006	0.3137	0.5661	0.0198	0.1208	0.4180	0.2021	0.3480
	[0.087,0.316]	[-0.119,0.038]	[-0.127,0.070]	[-0.229,-0.020]	[-0.031,0.265]	[-0.125,0.052]	[-0.071,0.015]	[-0.189,0.067]
Other	0.063	0.003	-0.076**	-0.072*	0.160***	-0.008	-0.028*	-0.042
	0.1188	0.9236	0.0293	0.0530	0.0025	0.8031	0.0677	0.3541
	[-0.016,0.143]	[-0.052,0.057]	[-0.145,-0.008]	[-0.145,0.001]	[0.057,0.263]	[-0.069,0.054]	[-0.058,0.002]	[-0.131,0.047]
Constant	0.108***	0.064***	0.124***	0.148***	0.240***	0.084***	0.028***	0.204***
	0.0000	0.0000	0.0000	0.0000	0.0000	0.0000	0.0008	0.0000
	[0.065,0.151]	[0.034,0.094]	[0.087,0.161]	[0.108,0.188]	[0.184,0.296]	[0.051,0.117]	[0.012,0.044]	[0.155,0.253]
Observations	397	397	397	397	397	397	397	397
R^2	0.031	0.003	0.012	0.019	0.025	0.002	0.011	0.004

Notes: Values of b show the estimated coefficients of linear probability models on the likelihood that the respective Species were detected, together with their p-value and confidence interval (95%). Baseline category for Residency is Germany. Statistical significance level * $p < 0.10$, ** $p < 0.05$, *** $p < 0.01$.

Table B10: Likelihood of colonization with carbapenemase-encoding Species for different Ethnicity - linear regression analysis

	A.baumannii b/p-value/CI95	Citrobact b/p-value/CI95	E.coli b/p-value/CI95	Enterobact b/p-value/CI95	K.pneumoniae b/p-value/CI95	Other Enterobact b/p-value/CI95	Other Nonfermenter b/p-value/CI95	P.aearuginosa b/p-value/CI95
Ethnicity								
Arabic	0.182***	-0.052	0.010	-0.149***	0.134*	-0.025	-0.029	-0.072
	0.0015	0.1868	0.8343	0.0043	0.0697	0.5739	0.1820	0.2562
	[0.070,0.294]	[-0.128,0.025]	[-0.087,0.107]	[-0.251,-0.047]	[-0.011,0.279]	[-0.111,0.062]	[-0.071,0.014]	[-0.197,0.053]
Kashmiri	0.052	-0.074	-0.103	-0.006	0.319*	0.073	-0.029	-0.231
	0.6966	0.4161	0.3715	0.9611	0.0652	0.4815	0.5680	0.1206
	[-0.210,0.314]	[-0.254,0.105]	[-0.330,0.124]	[-0.244,0.232]	[-0.020,0.659]	[-0.130,0.275]	[-0.128,0.071]	[-0.524,0.061]
Punjabi	0.222**	-0.074	-0.103	-0.149*	0.310***	-0.070	-0.029	-0.106
	0.0139	0.2273	0.1846	0.0689	0.0079	0.3119	0.3965	0.2887
	[0.045,0.398]	[-0.195,0.047]	[-0.256,0.050]	[-0.309,0.012]	[0.082,0.539]	[-0.207,0.066]	[-0.096,0.038]	[-0.303,0.091]
Somalian	0.144*	-0.074	0.132*	-0.149*	0.101	0.047	-0.029	-0.173*
	0.0983	0.2143	0.0815	0.0613	0.3728	0.4826	0.3831	0.0771
	[-0.027,0.316]	[-0.192,0.043]	[-0.017,0.281]	[-0.305,0.007]	[-0.121,0.323]	[-0.085,0.180]	[-0.094,0.036]	[-0.364,0.019]
Turkish	0.209**	-0.074	-0.053	-0.149**	0.198*	0.030	-0.029	-0.131
	0.0100	0.1805	0.4472	0.0436	0.0597	0.6345	0.3469	0.1463
	[0.050,0.368]	[-0.183,0.035]	[-0.191,0.084]	[-0.293,-0.004]	[-0.008,0.404]	[-0.093,0.153]	[-0.089,0.031]	[-0.309,0.046]
Other	0.066	0.024	-0.005	0.028	-0.036	0.067	-0.029	-0.114*
	0.2184	0.5197	0.9097	0.5694	0.6005	0.1065	0.1558	0.0578
	[-0.039,0.171]	[-0.049,0.096]	[-0.096,0.086]	[-0.068,0.123]	[-0.173,0.100]	[-0.014,0.148]	[-0.069,0.011]	[-0.231,0.004]
Constant	0.091***	0.074***	0.103***	0.149***	0.252***	0.070***	0.029***	0.231***
	0.0001	0.0000	0.0000	0.0000	0.0000	0.0001	0.0007	0.0000
	[0.047,0.135]	[0.044,0.104]	[0.065,0.141]	[0.109,0.189]	[0.195,0.309]	[0.036,0.104]	[0.012,0.046]	[0.182,0.280]
Observations	397	397	397	397	397	397	397	397
R^2	0.050	0.018	0.017	0.043	0.041	0.014	0.011	0.025

Notes: Values of b show the estimated coefficients of linear probability models on the likelihood that the respective Species were detected, together with their p-value and confidence interval (95%). Baseline category for Ethnicity is German. Statistical significance level * $p < 0.10$, ** $p < 0.05$, *** $p < 0.01$.

Table B11: Likelihood of Carbapenemases for different Residency - linear regression analysis

	GES<br>b/p-value/CI95	KPC<br>b/p-value/CI95	NDM<br>b/p-value/CI95	OXA-23<br>b/p-value/CI95	OXA-24-72<br>b/p-value/CI95	OXA-48<br>b/p-value/CI95	OXA-58<br>b/p-value/CI95	OXA-72<br>b/p-value/CI95	VIM<br>b/p-value/CI95
Residency									
Arabian Peninsula	0.032	-0.100**	-0.017	0.202***	-0.004	0.101	0.000	-0.008	-0.221***
	0.1507	0.0254	0.7859	0.0001	0.8099	0.2043	1.0000	0.6319	0.0032
	[-0.012,0.075]	[-0.188,-0.012]	[-0.143,0.108]	[0.098,0.305]	[-0.037,0.029]	[-0.055,0.257]	[-0.016,0.016]	[-0.041,0.025]	[-0.368,-0.075]
Other	-0.006	-0.043	-0.022	0.040	0.025**	0.144***	0.010	0.011	-0.183***
	0.6726	0.1688	0.6196	0.2784	0.0346	0.0097	0.1032	0.3431	0.0005
	[-0.037,0.024]	[-0.104,0.018]	[-0.110,0.065]	[-0.032,0.112]	[0.002,0.047]	[0.035,0.252]	[-0.002,0.021]	[-0.012,0.034]	[-0.285,-0.081]
Constant	0.016*	0.100***	0.184***	0.084***	0.004	0.304***	-0.000	0.008	0.364***
	0.0554	0.0000	0.0000	0.0000	0.5259	0.0000	1.0000	0.2070	0.0000
	[-0.000,0.032]	[0.067,0.133]	[0.136,0.232]	[0.045,0.123]	[-0.008,0.016]	[0.245,0.363]	[-0.006,0.006]	[-0.004,0.020]	[0.308,0.420]
Observations	397	397	397	397	397	397	397	397	397
R^2	0.007	0.015	0.001	0.036	0.012	0.018	0.007	0.003	0.043

Notes: Values of b show the estimated coefficients of linear probability models on the likelihood that the respective carbapenemases were detected, together with their p-value and confidence interval (95%). Baseline category for Residency is Germany. Statistical significance level * $p < 0.10$, ** $p < 0.05$, *** $p < 0.01$.

Table B12: Likelihood of Carbapenemases for different Ethnicity - linear regression analysis

	GES<br>b/p-value/CI95	KPC<br>b/p-value/CI95	NDM<br>b/p-value/CI95	OXA-23<br>b/p-value/CI95	OXA-24-72<br>b/p-value/CI95	OXA-48<br>b/p-value/CI95	OXA-58<br>b/p-value/CI95	OXA-72<br>b/p-value/CI95	VIM<br>b/p-value/CI95
Ethnicity									
Arabic	0.002	-0.103**	0.033	0.176***	-0.004	0.093	-0.004	0.023	-0.231***
	0.9243	0.0189	0.5951	0.0007	0.7999	0.2358	0.6181	0.1609	0.0017
	[-0.041,0.045]	[-0.189,-0.017]	[-0.089,0.155]	[0.075,0.276]	[-0.036,0.028]	[-0.061,0.247]	[-0.020,0.012]	[-0.009,0.055]	[-0.375,-0.088]
Kashmiri	-0.021	-0.103	-0.006	0.068	-0.004	0.090	-0.004	0.000	-0.082
	0.6846	0.3142	0.9676	0.5670	0.9137	0.6245	0.8312	1.0000	0.6319
	[-0.121,0.079]	[-0.305,0.098]	[-0.292,0.280]	[-0.167,0.303]	[-0.079,0.071]	[-0.270,0.450]	[-0.042,0.034]	[-0.074,0.074]	[-0.419,0.254]
Punjabi	-0.021	-0.103	0.039	0.176**	0.058**	0.099	-0.004	0.000	-0.180
	0.5464	0.1352	0.6925	0.0297	0.0235	0.4242	0.7516	1.0000	0.1185
	[-0.088,0.047]	[-0.239,0.032]	[-0.154,0.231]	[0.017,0.334]	[0.008,0.109]	[-0.144,0.341]	[-0.030,0.022]	[-0.050,0.050]	[-0.407,0.046]
Somalian	-0.021	-0.103	0.263***	0.043	0.055**	0.132	-0.004	0.000	-0.250**
	0.5349	0.1244	0.0060	0.5805	0.0289	0.2724	0.7447	1.0000	0.0261
	[-0.086,0.045]	[-0.235,0.029]	[0.076,0.450]	[-0.111,0.197]	[0.006,0.104]	[-0.104,0.367]	[-0.029,0.021]	[-0.049,0.049]	[-0.470,-0.030]
Turkish	-0.021	-0.053	-0.049	0.276***	-0.004	0.111	-0.004	0.000	-0.318***
	0.5034	0.3920	0.5808	0.0002	0.8583	0.3179	0.7254	1.0000	0.0024
	[-0.081,0.040]	[-0.176,0.069]	[-0.222,0.125]	[0.133,0.418]	[-0.050,0.041]	[-0.107,0.330]	[-0.027,0.019]	[-0.045,0.045]	[-0.522,-0.114]
Other	-0.001	-0.005	0.106*	-0.016	0.015	-0.104	-0.004	0.059***	-0.113
	0.9589	0.8983	0.0701	0.7462	0.3130	0.1603	0.5959	0.0001	0.1016
	[-0.041,0.039]	[-0.086,0.076]	[-0.009,0.221]	[-0.110,0.079]	[-0.015,0.046]	[-0.248,0.041]	[-0.019,0.011]	[0.029,0.089]	[-0.248,0.022]
Constant	0.021**	0.103***	0.149***	0.074***	0.004	0.339***	0.004	-0.000	0.368***
	0.0158	0.0000	0.0000	0.0002	0.5183	0.0000	0.2041	1.0000	0.0000
	[0.004,0.037]	[0.070,0.137]	[0.101,0.197]	[0.035,0.114]	[-0.008,0.017]	[0.278,0.399]	[-0.002,0.011]	[-0.012,0.012]	[0.311,0.424]
Observations	397	397	397	397	397	397	397	397	397
R^2	0.003	0.025	0.027	0.068	0.026	0.018	0.002	0.040	0.053

Notes: Values of b show the estimated coefficients of linear probability models on the likelihood that the respective carbapenemases were detected, together with their p-value and confidence interval (95%). Baseline category for Ethnicity is German. Statistical significance level * $p < 0.10$, ** $p < 0.05$, *** $p < 0.01$.

B.3 Logit Regressions

Table B13: Likelihood of colonization with carbapenemase-encoding Species for different Residency - marginal effects after logit regression

	A.baumannii b/p-value/CI95	Citrobact b/p-value/CI95	E.coli b/p-value/CI95	Enterobact b/p-value/CI95	K.pneumoniae b/p-value/CI95	Other Enterobact b/p-value/CI95	Other Nonfermenter b/p-value/CI95	P.aearuginosa b/p-value/CI95
Residency								
Arabian Peninsula	0.099*	-0.046	-0.012	-0.194*	0.088	-0.073	0.000	0.002
	0.0501	0.4552	0.8178	0.0693	0.2555	0.2258	.	0.9791
	[-0.000,0.199]	[-0.168,0.075]	[-0.117,0.092]	[-0.404,0.015]	[-0.064,0.239]	[-0.192,0.045]	[0.000,0.000]	[-0.143,0.146]
Other	-0.007	0.024	-0.084*	-0.051	0.111*	0.010	0.000	0.026
	0.8745	0.4713	0.0816	0.2828	0.0506	0.7852	.	0.6145
	[-0.090,0.076]	[-0.042,0.091]	[-0.179,0.011]	[-0.143,0.042]	[-0.000,0.223]	[-0.064,0.084]	[0.000,0.000]	[-0.075,0.127]
Control variables								
Age	-0.003***	0.001	0.001	0.000	-0.001	0.000	0.004	0.001
	0.0010	0.5040	0.3466	0.8521	0.5396	0.9077	0.1284	0.4726
	[-0.005,-0.001]	[-0.001,0.003]	[-0.001,0.003]	[-0.002,0.003]	[-0.004,0.002]	[-0.002,0.002]	[-0.001,0.008]	[-0.002,0.004]
Female	-0.022	-0.068*	0.051*	0.021	0.067	-0.001	-0.001	-0.066
	0.5733	0.0682	0.0975	0.5450	0.1756	0.9698	0.9812	0.1325
	[-0.099,0.055]	[-0.141,0.005]	[-0.009,0.112]	[-0.047,0.088]	[-0.030,0.163]	[-0.060,0.058]	[-0.089,0.087]	[-0.151,0.020]
Oncological:yes	-0.228***	0.034	0.013	-0.018	-0.134*	-0.011	0.131**	0.148***
	0.0076	0.3066	0.7412	0.6973	0.0639	0.7697	0.0402	0.0012
	[-0.396,-0.061]	[-0.031,0.100]	[-0.065,0.092]	[-0.109,0.073]	[-0.275,0.008]	[-0.087,0.064]	[0.006,0.255]	[0.059,0.237]
History of ICU:yes	0.069	0.071*	0.019	0.017	-0.071	-0.036	0.000	0.017
	0.3252	0.0961	0.7287	0.7937	0.4889	0.5490	.	0.8059
	[-0.069,0.207]	[-0.013,0.154]	[-0.090,0.129]	[-0.108,0.141]	[-0.271,0.129]	[-0.155,0.082]	[0.000,0.000]	[-0.117,0.151]
ICU:yes	-0.154**	-0.044	-0.018	0.056	0.056	0.078	0.000	-0.030
	0.0340	0.2811	0.7409	0.3533	0.5811	0.1896	.	0.6642
	[-0.297,-0.012]	[-0.123,0.036]	[-0.128,0.091]	[-0.063,0.175]	[-0.144,0.256]	[-0.038,0.194]	[0.000,0.000]	[-0.166,0.106]
Days hospitalised	-0.001*	-0.000	-0.001	-0.000	-0.000	0.001**	-0.000	0.001**
	0.0567	0.4039	0.2003	0.5667	0.3767	0.0165	0.9711	0.0189
	[-0.002,0.000]	[-0.001,0.000]	[-0.001,0.000]	[-0.001,0.001]	[-0.002,0.001]	[0.000,0.001]	[-0.001,0.001]	[0.000,0.001]
Observations	390	390	390	390	390	390	118	390

Notes: Values of b show the estimated marginal effects of logit models on the likelihood that the respective Species were detected, together with their p-value and confidence interval (95%). Baseline category for Residency is Germany. Statistical significance level * $p < 0.10$, ** $p < 0.05$, *** $p < 0.01$.

Table B14: Likelihood of colonization with carbapenemase-encoding Species for different Ethnicity - marginal effects after logit regression

	A.baumannii b/p-value/CI95	Citrobact b/p-value/CI95	E.coli b/p-value/CI95	Enterobact b/p-value/CI95	K.pneumoniae b/p-value/CI95	Other Enterobact b/p-value/CI95	Other Nonfermenter b/p-value/CI95	P.aearuginosa b/p-value/CI95
Ethnicity								
Arabic	0.123**	-0.070	-0.000	0.000	0.096	-0.038	0.000	-0.003
	0.0131	0.3243	0.9965	.	0.1850	0.5218	.	0.9575
	[0.026,0.220]	[-0.209,0.069]	[-0.109,0.108]	[0.000,0.000]	[-0.046,0.237]	[-0.155,0.079]	[0.000,0.000]	[-0.131,0.124]
Kashmiri	-0.004	0.000	0.000	0.007	0.237	0.083	0.000	0.000
	0.9735	.	.	0.9598	0.1167	0.3197	.	.
	[-0.248,0.240]	[0.000,0.000]	[0.000,0.000]	[-0.273,0.288]	[-0.059,0.533]	[-0.080,0.246]	[0.000,0.000]	[0.000,0.000]
Punjabi	0.085	0.000	0.000	0.000	0.247**	0.000	0.000	-0.088
	0.2337	.	.	.	0.0204	.	.	0.4778
	[-0.055,0.224]	[0.000,0.000]	[0.000,0.000]	[0.000,0.000]	[0.038,0.455]	[0.000,0.000]	[0.000,0.000]	[-0.329,0.154]
Somalian	0.104	0.000	0.112*	0.000	0.101	0.062	0.000	-0.201
	0.1730	.	0.0677	.	0.3465	0.3225	.	0.1582
	[-0.045,0.253]	[0.000,0.000]	[-0.008,0.232]	[0.000,0.000]	[-0.109,0.312]	[-0.061,0.184]	[0.000,0.000]	[-0.481,0.078]
Turkish	0.091	0.000	-0.051	0.000	0.153	0.055	0.000	-0.093
	0.1553	.	0.6059	.	0.1088	0.3680	.	0.3814
	[-0.035,0.218]	[0.000,0.000]	[-0.244,0.142]	[0.000,0.000]	[-0.034,0.341]	[-0.064,0.174]	[0.000,0.000]	[-0.302,0.115]
Other	0.026	0.026	0.003	0.032	-0.053	0.070*	0.000	-0.106
	0.6162	0.4908	0.9504	0.5694	0.4792	0.0680	.	0.1330
	[-0.077,0.129]	[-0.048,0.100]	[-0.094,0.100]	[-0.078,0.141]	[-0.200,0.094]	[-0.005,0.146]	[0.000,0.000]	[-0.244,0.032]
Control variables								
Age	-0.003***	0.000	0.001	0.000	-0.000	0.001	0.004*	0.000
	0.0036	0.6878	0.2953	0.9871	0.7357	0.6193	0.0580	0.9593
	[-0.005,-0.001]	[-0.002,0.003]	[-0.001,0.004]	[-0.003,0.003]	[-0.003,0.002]	[-0.002,0.003]	[-0.000,0.008]	[-0.003,0.003]
Female	-0.012	-0.090**	0.050	0.015	0.080	-0.006	0.022	-0.064
	0.7613	0.0443	0.1298	0.7362	0.1001	0.8382	0.5816	0.1427
	[-0.090,0.066]	[-0.177,-0.002]	[-0.015,0.115]	[-0.073,0.104]	[-0.015,0.176]	[-0.068,0.055]	[-0.055,0.099]	[-0.151,0.022]
Oncological:yes	-0.222***	0.022	0.019	-0.015	-0.149**	-0.022	0.173***	0.132***
	0.0088	0.5430	0.6547	0.7934	0.0323	0.5835	0.0045	0.0021
	[-0.388,-0.056]	[-0.049,0.094]	[-0.063,0.101]	[-0.131,0.100]	[-0.285,-0.013]	[-0.099,0.056]	[0.054,0.293]	[0.048,0.216]
History of ICU:yes	0.086	0.059	0.050	0.024	-0.101	-0.048	0.000	0.001
	0.1953	0.1848	0.3873	0.7621	0.2982	0.4303	.	0.9846
	[-0.044,0.216]	[-0.028,0.145]	[-0.064,0.164]	[-0.130,0.177]	[-0.292,0.089]	[-0.166,0.071]	[0.000,0.000]	[-0.128,0.130]
ICU:yes	-0.157**	-0.050	-0.017	0.078	0.063	0.093	0.000	-0.044
	0.0294	0.2890	0.7751	0.3164	0.5351	0.1279	.	0.5329
	[-0.298,-0.016]	[-0.143,0.043]	[-0.134,0.100]	[-0.075,0.232]	[-0.136,0.261]	[-0.027,0.214]	[0.000,0.000]	[-0.182,0.094]
Days hospitalised	-0.001*	-0.000	-0.001	-0.000	-0.000	0.001***	0.000	0.001**
	0.0821	0.3634	0.2071	0.4897	0.4377	0.0083	0.8265	0.0192
	[-0.002,0.000]	[-0.001,0.000]	[-0.001,0.000]	[-0.001,0.001]	[-0.001,0.001]	[0.000,0.001]	[-0.001,0.001]	[0.000,0.001]
Observations	390	330	367	295	390	374	135	383

Notes: Values of b show the estimated marginal effects of logit models on the likelihood that the respective Species were detected, together with their p-value and confidence interval (95%). Baseline category for Ethnicity is German. Statistical significance level * $p < 0.10$, ** $p < 0.05$, *** $p < 0.01$.

Table B15: Likelihood of Carbapenemases for different Residency - marginal effects after logit regression

	GES<br>b/p-value/CI95	KPC<br>b/p-value/CI95	NDM<br>b/p-value/CI95	OXA-23<br>b/p-value/CI95	OXA-24-72<br>b/p-value/CI95	OXA-48<br>b/p-value/CI95	OXA-58<br>b/p-value/CI95	OXA-72<br>b/p-value/CI95	VIM<br>b/p-value/CI95
Residency									
Arabian Peninsula	0.035*	0.000	-0.011	0.106**	0.000	0.066	0.000	0.000	-0.192**
	0.0742	.	0.8774	0.0172	.	0.4221	.	.	0.0350
	[-0.003,0.074]	[0.000,0.000]	[-0.146,0.125]	[0.019,0.194]	[0.000,0.000]	[-0.094,0.226]	[0.000,0.000]	[0.000,0.000]	[-0.370,-0.014]
Other	-0.010	0.055	0.004	-0.009	0.048*	0.062	0.000	0.001	-0.093
	0.6136	0.3567	0.9335	0.8221	0.0698	0.3007	.	0.9636	0.1267
	[-0.051,0.030]	[-0.062,0.171]	[-0.097,0.106]	[-0.088,0.070]	[-0.004,0.100]	[-0.056,0.181]	[0.000,0.000]	[-0.044,0.046]	[-0.212,0.026]
Control variables									
Age	0.000	0.001	0.001	-0.002*	0.000	-0.001	0.000	-0.001*	0.002
	0.2349	0.4879	0.2965	0.0617	0.8261	0.3928	.	0.0607	0.2781
	[-0.000,0.001]	[-0.002,0.004]	[-0.001,0.004]	[-0.003,0.000]	[-0.002,0.002]	[-0.004,0.002]	[0.000,0.000]	[-0.002,0.000]	[-0.001,0.005]
Female	0.007	-0.002	0.070*	-0.051	0.038	0.089*	0.000	-0.001	-0.120**
	0.5971	0.9497	0.0775	0.1860	0.1143	0.0790	.	0.9803	0.0145
	[-0.019,0.033]	[-0.080,0.075]	[-0.008,0.147]	[-0.125,0.024]	[-0.009,0.085]	[-0.010,0.189]	[0.000,0.000]	[-0.052,0.051]	[-0.217,-0.024]
Oncological:yes	0.020	0.000	0.120**	-0.161**	0.000	-0.146**	0.000	0.000	0.229***
	0.2754	.	0.0125	0.0291	.	0.0393	.	.	0.0000
	[-0.016,0.056]	[0.000,0.000]	[0.026,0.214]	[-0.306,-0.016]	[0.000,0.000]	[-0.284,-0.007]	[0.000,0.000]	[0.000,0.000]	[0.125,0.334]
History of ICU:yes	-0.274***	0.229***	0.019	0.014	0.000	-0.257**	0.000	0.000	0.014
	0.0033	0.0005	0.8041	0.8432	.	0.0171	.	.	0.8613
	[-0.457,-0.091]	[0.099,0.360]	[-0.128,0.165]	[-0.122,0.149]	[0.000,0.000]	[-0.468,-0.046]	[0.000,0.000]	[0.000,0.000]	[-0.146,0.174]
ICU:yes	0.257***	-0.048	-0.039	-0.052	0.000	0.138	0.000	0.000	0.070
	0.0030	0.3067	0.6122	0.4600	.	0.2092	.	.	0.3852
	[0.088,0.427]	[-0.140,0.044]	[-0.188,0.111]	[-0.189,0.086]	[0.000,0.000]	[-0.078,0.354]	[0.000,0.000]	[0.000,0.000]	[-0.088,0.229]
Days hospitalised	-0.000	-0.000	-0.000	-0.001	-0.001	0.001*	0.000	-0.001	0.000
	0.2856	0.8170	0.3542	0.1930	0.2850	0.0538	.	0.3728	0.5372
	[-0.001,0.000]	[-0.001,0.001]	[-0.001,0.000]	[-0.002,0.000]	[-0.003,0.001]	[-0.000,0.002]	[0.000,0.000]	[-0.004,0.001]	[-0.001,0.001]
Observations	390	287	390	390	174	390	76	174	390

Notes: Values of b show the estimated marginal effects of logit models on the likelihood that the respective carbapenemases were detected, together with their p-value and confidence interval (95%). Baseline category for Residency is Germany. Statistical significance level * $p < 0.10$, ** $p < 0.05$, *** $p < 0.01$.

Table B16: Likelihood of Carbapenemases for different Ethnicity - marginal effects after logit regression

	GES<br>b/p-value/CI95	KPC<br>b/p-value/CI95	NDM<br>b/p-value/CI95	OXA-23<br>b/p-value/CI95	OXA-24-72<br>b/p-value/CI95	OXA-48<br>b/p-value/CI95	OXA-58<br>b/p-value/CI95	OXA-72<br>b/p-value/CI95	VIM<br>b/p-value/CI95
Ethnicity									
Arabic	0.013	0.000	0.061	0.115***	0.000	0.033	0.000	-0.066	-0.196**
	0.5892	.	0.3379	0.0080	.	0.6770	.	0.4746	0.0230
	[-0.034,0.060]	[0.000,0.000]	[-0.064,0.186]	[0.030,0.200]	[0.000,0.000]	[-0.121,0.186]	[0.000,0.000]	[-0.248,0.115]	[-0.365,-0.027]
Kashmiri	0.000	0.000	0.038	0.030	0.000	0.039	0.000	0.000	0.017
	.	.	0.8038	0.7750	.	0.8171	.	.	0.9126
	[0.000,0.000]	[0.000,0.000]	[-0.260,0.335]	[-0.175,0.235]	[0.000,0.000]	[-0.290,0.368]	[0.000,0.000]	[0.000,0.000]	[-0.283,0.317]
Punjabi	0.000	0.000	0.108	0.071	0.302	0.023	0.000	0.000	-0.108
	.	.	0.2653	0.2668	0.6965	0.8457	.	.	0.3835
	[0.000,0.000]	[0.000,0.000]	[-0.082,0.297]	[-0.054,0.196]	[-1.217,1.821]	[-0.210,0.256]	[0.000,0.000]	[0.000,0.000]	[-0.352,0.135]
Somalian	0.000	0.000	0.194**	0.027	0.348	0.083	0.000	0.000	-0.246*
	.	.	0.0119	0.7299	0.6478	0.4665	.	.	0.0795
	[0.000,0.000]	[0.000,0.000]	[0.043,0.345]	[-0.127,0.181]	[-1.145,1.841]	[-0.140,0.305]	[0.000,0.000]	[0.000,0.000]	[-0.521,0.029]
Turkish	0.000	-0.023	-0.022	0.130**	0.000	0.075	0.000	0.000	-0.402**
	.	0.8351	0.8394	0.0131	.	0.4821	.	.	0.0311
	[0.000,0.000]	[-0.238,0.192]	[-0.233,0.189]	[0.027,0.232]	[0.000,0.000]	[-0.134,0.284]	[0.000,0.000]	[0.000,0.000]	[-0.767,-0.037]
Other	0.008	0.018	0.099*	-0.049	0.143*	-0.140*	0.000	0.000	-0.052
	0.7395	0.7509	0.0693	0.4241	0.0871	0.0779	.	.	0.4391
	[-0.038,0.054]	[-0.094,0.130]	[-0.008,0.206]	[-0.170,0.072]	[-0.021,0.306]	[-0.296,0.016]	[0.000,0.000]	[0.000,0.000]	[-0.184,0.080]
Control variables									
Age	0.000	0.000	0.002*	-0.002*	-0.001	-0.002	-0.000	-0.004*	0.001
	0.4629	0.7845	0.0857	0.0755	0.8673	0.2533	.	0.0861	0.3858
	[-0.001,0.001]	[-0.003,0.004]	[-0.000,0.005]	[-0.003,0.000]	[-0.010,0.008]	[-0.005,0.001]	[-0.000,-0.000]	[-0.008,0.001]	[-0.002,0.005]
Female	0.014	-0.008	0.066*	-0.038	0.328	0.095*	0.000	-0.008	-0.120**
	0.4088	0.8519	0.0960	0.3211	0.6684	0.0646	.	0.9354	0.0135
	[-0.019,0.046]	[-0.097,0.080]	[-0.012,0.143]	[-0.114,0.037]	[-1.172,1.828]	[-0.006,0.195]	[0.000,0.000]	[-0.190,0.175]	[-0.214,-0.025]
Oncological:yes	0.027	0.000	0.116**	-0.144**	0.000	-0.164**	0.000	0.000	0.252***
	0.2088	.	0.0119	0.0440	.	0.0156	.	.	0.0000
	[-0.015,0.069]	[0.000,0.000]	[0.026,0.207]	[-0.284,-0.004]	[0.000,0.000]	[-0.298,-0.031]	[0.000,0.000]	[0.000,0.000]	[0.153,0.350]
History of ICU:yes	-0.268	0.212***	0.031	0.035	0.000	-0.279***	0.000	0.000	0.037
	0.9928	0.0004	0.6594	0.5958	.	0.0071	.	.	0.6326
	[-58.668,58.131]	[0.094,0.331]	[-0.108,0.170]	[-0.094,0.164]	[0.000,0.000]	[-0.483,-0.076]	[0.000,0.000]	[0.000,0.000]	[-0.115,0.190]
ICU:yes	0.248	-0.052	-0.034	-0.061	0.000	0.141	0.000	0.000	0.059
	0.9934	0.3191	0.6517	0.3724	.	0.1994	.	.	0.4644
	[-58.151,58.647]	[-0.155,0.051]	[-0.182,0.114]	[-0.197,0.074]	[0.000,0.000]	[-0.075,0.357]	[0.000,0.000]	[0.000,0.000]	[-0.099,0.216]
Days hospitalised	-0.001	-0.000	-0.000	-0.001	-0.006	0.001**	0.000	-0.003	0.000
	0.2310	0.9146	0.3494	0.2845	0.2204	0.0438	.	0.4559	0.8371
	[-0.001,0.000]	[-0.001,0.001]	[-0.001,0.000]	[-0.002,0.000]	[-0.015,0.003]	[0.000,0.002]	[0.000,0.000]	[-0.010,0.005]	[-0.001,0.001]
Observations	330	252	390	390	153	390	70	45	390

Notes: Values of b show the estimated marginal effects of logit models on the likelihood that the respective carbapenemases were detected, together with their p-value and confidence interval (95%). Baseline category for Ethnicity is German. Statistical significance level * $p < 0.10$, ** $p < 0.05$, *** $p < 0.01$.
