# Supplementary material for: Global Distribution Patterns of Carbapenemase-Encoding Bacteria in a New Light: Clues on a Role for Ethnicity
Source: Front Cell Infect Microbiol. 2021 Jun 29;11:659753. doi: 10.3389/fcimb.2021.659753 (PMC8276097; doi:10.3389/fcimb.2021.659753)
Supplement: Supplementary file 2 [file DataSheet_2.pdf]

Appendix C

Tables

Table C1: Table summarizing sample date, MLST-type, ward and acquired resistance genes conferring decreased susceptibility against beta-lactam class antibiotics of sequenced KPC-encoding *Enterobacter cloacae* complex isolates.

**Isolate**	**Sample Date**	**MLST**	**Ward**	**BLRG**
Enterobacter cloacae complex	15.05.2019	165	A	blaTEM-1A ; blaMIR-1, -5, -6 ; blaOXA-9, -10; blaKPC-2
Enterobacter cloacae complex	26.06.2019	419	B	blaCTX-M-9 ; blaTEM-1B ; blaOXA-1 ; blaKPC-2
Enterobacter cloacae complex	30.06.2019	419	C	blaCTX-M-9 ; blaTEM-1B ; blaOXA-1 ; blaKPC-2
Enterobacter cloacae complex	06.07.2019	419	D	blaCTX-M-9 ; blaTEM-1B ; blaACT-14 ; blaOXA-1 ; blaKPC-2
Enterobacter cloacae complex	12.07.2019	419	D	blaCTX-M-9 ; blaTEM-1B ; blaACT-14 ; blaOXA-1 ; blaKPC-2
Enterobacter cloacae complex	14.07.2019	419	B	blaCTX-M-9 ; blaTEM-1B ; blaDHA-12 blaACT-14 ; blaOXA-1 ; blaKPC-2
Enterobacter cloacae complex	20.07.2019	419	E	blaCTX-M-9 ; blaTEM-1B ; blaACT-14 ; blaOXA-1 ; blaKPC-2
Enterobacter cloacae complex	21.07.2019	419	D	blaCTX-M-9 ; blaTEM-1B ; blaACT-14 ; blaOXA-1 ; blaKPC-2
Enterobacter cloacae complex	21.07.2019	419	D	blaCTX-M-9 ; blaTEM-1B ; blaACT-14 ; blaOXA-1 ; blaKPC-2
Enterobacter cloacae complex	01.08.2019	419	F	blaCTX-M-9 ; blaTEM-1B ; blaACT-14 ; blaOXA-1 ; blaKPC-2

Table C2: Table summarizing sample date, MLST-type, ward and OXA variant of sequenced OXA-48-like encoding Klebsiella pneumoniae isolates.

**Isolate**	**Sample Date**	**MLST**	**OXA Variant**	**Ward**
Klebsiella pneumoniae	02.01.2019	466	48	G
Klebsiella pneumoniae	25.01.2019	101	181/32	H
Klebsiella pneumoniae	25.01.2019	395	48	I
Klebsiella pneumoniae	28.01.2019	16	181/32	J
Klebsiella pneumoniae	20.02.2019	16	181/32	K
Klebsiella pneumoniae	27.02.2019	1198	48	L
Klebsiella pneumoniae	15.04.2019	16	48	M
Klebsiella pneumoniae	13.05.2019	78	48	O
Klebsiella pneumoniae	16.07.2019	48	48	D
Klebsiella pneumoniae	17.08.2019	14	48	P
Klebsiella pneumoniae	16.10.2019	3440	48	B
Klebsiella pneumoniae	25.10.2019	2096	232	L
